# Probing the signaling requirements for naive human pluripotency by high-throughput chemical screening

**DOI:** 10.1016/j.celrep.2021.109233

**Published:** 2021-06-15

**Authors:** Shafqat A. Khan, Kyoung-mi Park, Laura A. Fischer, Chen Dong, Tenzin Lungjangwa, Marta Jimenez, Dominick Casalena, Brian Chew, Sabine Dietmann, Douglas S. Auld, Rudolf Jaenisch, Thorold W. Theunissen

**Affiliations:** 1 Department of Developmental Biology and Center of Regenerative Medicine, Washington University School of Medicine, St. Louis, MO 63110, USA; 2 Whitehead Institute for Biomedical Research, Cambridge, MA 02142, USA; 3 Novartis Institutes for Biomedical Research, Cambridge, MA 02139, USA; 4 Department of Biology, Massachusetts Institute of Technology, Cambridge, MA 02142, USA; 5 Lead contact

## Abstract

Naive human embryonic stem cells (hESCs) have been isolated that more closely resemble the pre-implantation epiblast compared to conventional “primed” hESCs, but the signaling principles underlying these discrete stem cell states remain incompletely understood. Here, we describe the results from a high-throughput screen using ~3,000 well-annotated compounds to identify essential signaling requirements for naive human pluripotency. We report that MEK1/2 inhibitors can be replaced during maintenance of naive human pluripotency by inhibitors targeting either upstream (FGFR, RAF) or downstream (ERK1/2) kinases. Naive hESCs maintained under these alternative conditions display elevated levels of ERK phosphorylation but retain genome-wide DNA hypomethylation and a transcriptional identity of the pre-implantation epiblast. In contrast, dual inhibition of MEK and ERK promotes efficient primed-to-naive resetting in combination with PKC, ROCK, and TNKS inhibitors and activin A. This work demonstrates that induction and maintenance of naive human pluripotency are governed by distinct signaling requirements.

## In brief

Khan et al. describe a high-throughput chemical screen to identify essential signaling requirements for naive human pluripotency in minimal conditions. They report that naive hESCs can be maintained by blocking distinct nodes in the FGF signaling pathway and that dual MEK/ERK inhibition promotes efficient primed-to-naive resetting in combination with activin A.

## Graphical abstract

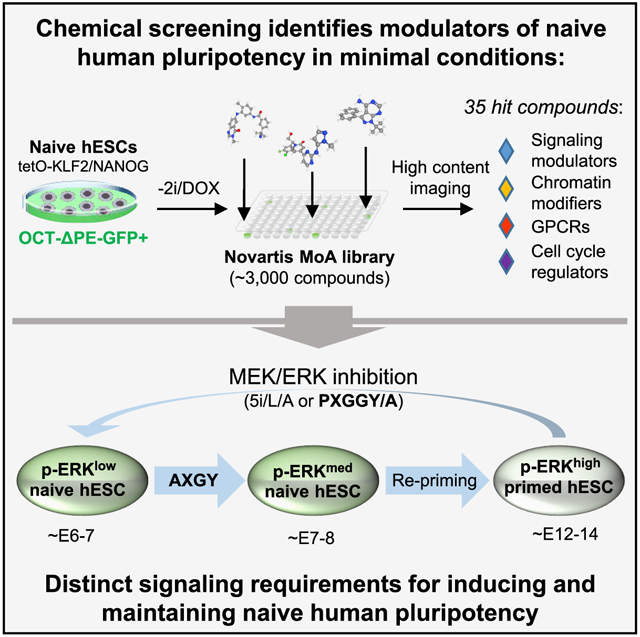

## INTRODUCTION

A major objective in stem cell research is to devise *in vitro* culture conditions for pluripotent stem cells (PSCs) that recapitulate specific stages of embryonic development. The use of MEK and GSK3 inhibitors and leukemia inhibitory factor (2i/LIF) captures mouse embryonic stem cells (ESCs) in a “naive” state of pluripotency that closely corresponds to the pre-implantation epiblast at embryonic day (E) 4.5 (Boroviak et al., 2015; Ying et al., 2008). This naive state of pluripotency contrasts with the “primed” pluripotent state observed in mouse epiblast stem cells (EpiSCs), which aligns more closely with the anterior primitive streak of the late-gastrula stage embryo (Brons et al., 2007; Kojima et al., 2014; Tesar et al., 2007). Overlapping biological and molecular features between human PSCs (hPSCs) and mouse EpiSCs lend support to the notion that hPSCs adopt a primed pluripotent identity when derived under conventional conditions (Nichols and Smith, 2009). Indeed, transcriptome profiling of primate embryos confirmed that conventional hPSCs most closely correlate with the late post-implantation epiblast (Nakamura et al., 2016). Nevertheless, recent work indicates that a subpopulation of conventional hPSCs with high self-renewal capacity displays properties more aligned with the early post-implantation epiblast (Cornacchia et al., 2019; Lau et al., 2020). Conventional hPSCs also exhibit some primate-specific features that are not observed in either mouse ESCs or EpiSCs, such as expression of N-cadherin at colony boundaries (Nakanishi et al., 2019).

Over the past decade, a number of groups have attempted to induce features of naive pluripotency in hPSCs using chemical and genetic approaches (Chan et al., 2013; Gafni et al., 2013; Hanna et al., 2010; Takashima et al., 2014; Theunissen et al., 2014; Ware et al., 2014; Zimmerlin et al., 2016). Based on comparisons to single cell RNA sequencing (scRNA-seq) data from human and non-human primate embryos (Huang et al., 2014; Nakamura et al., 2016; Stirparo et al., 2018), naive cells derived in two specific culture conditions display particularly strong transcriptional signatures of the pre-implantation embryo: t2i/L/Gö, which consists of titrated 2i/LIF and a PKC inhibitor (Takashima et al., 2014), and 5i/L/A, which consists of MEK, GSK3, BRAF, SRC, and ROCK inhibitors together with LIF and activin A (Theunissen et al., 2014). Naive hPSCs have provided a cellular model system to investigate human-specific mechanisms of X chromosome regulation (Sahakyan et al., 2017; Vallot et al., 2017) and the role of transposable elements (TEs) that are associated with early embryogenesis (Pontis et al., 2019; Theunissen et al., 2016). In addition, recent findings indicate that these cells also harbor the ability to acquire extraembryonic fates (Cinkornpumin et al., 2020; Dong et al., 2020; Guo et al., 2021; Io et al., 2021; Linneberg-Agerholm et al., 2019) and give rise to human blastocyst-like structures (Yu et al., 2021).

Despite the progress cited above, important questions remain about the nature of human pluripotent states and the utility of naive hPSCs in regenerative medicine. Naive hPSCs derived in t2i/L/Gö or 5i/L/A exhibit a global loss of imprinting and extended culture in 5i/L/A leads to genomic instability (Pastor et al., 2016; Theunissen et al., 2016). This has been attributed to global DNA hypomethylation resulting from the use of a MEK1/2 inhibitor, PD0325901, which is a near-universal component of naive stem cell protocols described to date (Choi et al., 2017; Yagi et al., 2017). While a SRC inhibitor could replace the MEK inhibitor in mouse ESCs, it was unable to do so in naive hPSCs (Choi et al., 2017; Yagi et al., 2017). In addition, titration of MEK inhibition was reported to enhance the genomic stability of naive hPSCs in 5i/L/A but was not tolerated in t2i/L/Gö, suggesting that MEK inhibition is essential for the naive state of human pluripotency (Di Stefano et al., 2018). Here, we performed high-throughput chemical screening to discover alternative compounds that can maintain naive human pluripotency in the absence of MEK1/2 inhibitors. We also omitted GSK3 inhibition as naive hPSCs cultured in the absence of a GSK3 inhibitor maintained a naive-specific transcriptome (Theunissen et al., 2016). This work uncovers distinct signaling requirements for induction and maintenance of naive human pluripotency and provides a basis for refinement of naive culture regimes.

## RESULTS

### High-throughput screening identifies modulators of naive human pluripotency in minimal conditions

**Figure 1.**
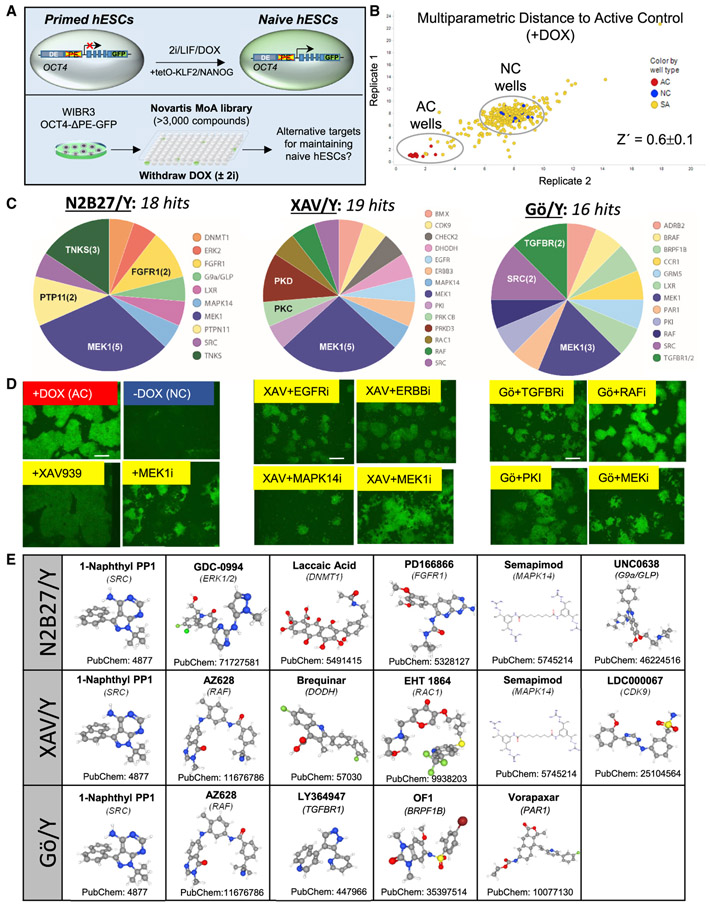
High-throughput chemical screening for modulators of naive human pluripotency in minimal conditions (A) Experimental strategy for identifying compounds that maintain naive human pluripotency in the presence or absence of MEK1/2 and GSK3 inhibitors (2i) using WIBR3 OCT4-ΔPE-GFP^+^ naive hESCs (Theunissen et al., 2014). (B) Multi-parametric data analysis (MPDA) from a representative 384-well plate analyzed in two biological replicates showing the activity of small molecules with respect to active controls (AC, +DOX) and negative controls (NC, −DOX) based on high-content imaging. (C) Pie charts summarizing the target classes of validated hit compounds in N2B27 medium supplemented with Y-27632 alone (N2B27/Y) (left), XAV939 and Y-27632 (XAV/Y) (middle), and Gö6983 and Y-27632 (Gö/Y) (right). Hit compounds were validated in two biological replicates. The scale bar depicts 260 μm. (D) Fluorescent images of active control (+DOX), negative control (−DOX), and selected hit compounds in N2B27/Y (left), XAV/Y (middle), and Gö/Y (right). Hit compounds were validated in two biological replicates. (E) Structures and CIDs of 13 commercially available hit compounds that displayed validated activity in maintaining naive human pluripotency in the three examined basal media in the absence of 2i (Y, Gö/Y, and XAV/Y). Source: PubChem. A full list of validated hit compounds in the absence of 2i is included in Table S2. See also Figure S1 and Table S2.

Our prior study, which resulted in the identification of the 5i/L/A cocktail, screened a library of 230 kinase inhibitors to identify compounds that can sustain naive-specific reporter activity in combination with 2i/LIF (Theunissen et al., 2014). Here, we considered whether alternative modulators of naive human pluripotency may be identified by repeating this screen on a high-throughput scale and applying a dynamic chemogenetic library of ~3,000 chemical probes in which a well-defined target is known for each of the compounds (Novartis Mechanism of Action Box) (Canham et al., 2020) either in the presence or absence of 2i. Similar to our prior study, we used naive hESCs that were generated with doxycycline (Dox)-inducible transgenes driving exogenous KLF2 and NANOG transgenes (Theunissen et al., 2014). These cells contain an endogenous *OCT4* reporter allele in which the primed-specific proximal enhancer has been deleted (*OCT4-ΔPE-GFP*). Upon withdrawal of DOX, GFP reporter activity was reduced and colony morphology was lost within 5 days, providing a convenient time window for high-throughput screening (Figure 1A).

As a pilot experiment, we seeded 3,000 OCT4-ΔPE-GF P^+^-naive hESCs per well in 384-well plates and removed DOX but continued to culture the cells in the presence of 2i and the Rho-associated kinase (ROCK) inhibitor Y-27632, which promotes viability after single cell dissociation (Watanabe et al., 2007) (2i/Y). We omitted LIF since its removal from established naive hESCs had little impact on naive-specific enhancer activity and gene expression (Theunissen et al., 2014). Individual compounds were applied on days 1 and 4, and images were obtained on day 5. We designed a multi-parametric data analysis (MPDA) algorithm to analyze these images, computing a Mahalanobis distance score between each compound and the active (+DOX) and negative (−DOX) control wells based on features that included area, compactness, and fluorescence intensity of individual object regions (Figure 1B) (see STAR Methods for details). This enabled us to distinguish objects that were likely due to cell death and phenotypes that were disparate from the desired phenotype observed in the positive control wells (Figure S1A). The screen showed good performance (Z′ = 0.6 ± 0.1), and hits were called based on distance to the active control (+DOX) wells. This screen identified 33 validated hit compounds that could synergize with the 2i cocktail in maintaining naive colony morphology and reporter activity upon removal of DOX-inducible transgenes (Figures S1B and S1C; Table S1). These hits included several target classes that have previously been implicated in control of naive human pluripotency, such as PKC (Takashima et al., 2014), BRAF, FGFR, VEGFR (Theunissen et al., 2014), and p38 MAPK (Gafni et al., 2013), demonstrating the capability of our screen to identify chemical modulators of naive human pluripotency.

We investigated whether our screening platform could be adapted to discover alternative compounds that sustain naive human pluripotency in the absence of 2i. OCT4-ΔPE-GF P^+^-naive hESCs were again seeded in 384-well plates, but this time compounds were supplemented only with Y-27632 (N2B27/Y). Pilot assays revealed that a maximal Z′factor was obtained in absence of 2i by performing image acquisition on day 7 post-DOX withdrawal.

As expected, MEK1 inhibitors emerged as the top-ranked category during validation assays (28%). In addition, a robust increase in GFP activity was confirmed in presence of several tankyrase (TNKS) inhibitors, as well as inhibitors of ERK, DNMT1, FGFR1, and SRC (Figures 1C and 1D, left panel). The TNKS inhibitor XAV939, which showed a particularly strong effect, inhibits WNT signaling and was shown to be beneficial for naive human pluripotency in two studies prior to this screen (Guo et al., 2017; Zimmerlin et al., 2016). Compared to inhibition of MEK1, treatment with XAV939 yielded larger colonies but a slightly dimmer OCT4-ΔPE-GFP signal (Figure 1D, left panel).

We then re-screened the Mechanism of Action library to identify compounds that could synergize with both XAV939 and Y-27632 (XAV/Y). MEK1 again appeared as the top target class, but we also identified and validated other hit compounds that could synergize with TNKS inhibition, including inhibitors of EGFR, PKC, RAF, and SRC (Figures 1C and 1D, middle panel). PKC was an enriched target class in both our 2i/Y and XAV/Y screens (Figure 1C; Figures S1B and S1C), which is consistent with inclusion of the PKC inhibitor Gö6983 in the t2i/L/Gö formulation (Takashima et al., 2014). We therefore also re-screened the Mechanism of Action library in the presence of the PKC inhibitor Gö6983 and Y-27632 (Go/Y). This screen identified synergy between Gö6983 and inhibitors of RAF, TGFBR2, SRC, and other targets (Figures 1C and 1D, right panel). Excluding MEK1/2 inhibitors, our high-throughput screens identified 35 hit compounds that showed activity in maintaining naive human pluripotency in one or several of the three examined basal conditions (Y, Gö/Y and XAV/Y) (Table S2). These compounds included inhibitors of signal transduction cascades, G-protein-coupled receptors, chromatin modifiers, cell-cycle regulators, and other targets. In cases where multiple hit compounds converged on the same molecular target (RAF, SRC, TGFBR, and TNKS), the compound with greater activity was selected for follow-up experiments.

### Defining the activity of selected hit compounds during extended naive hPSC culture

**Figure 2.**
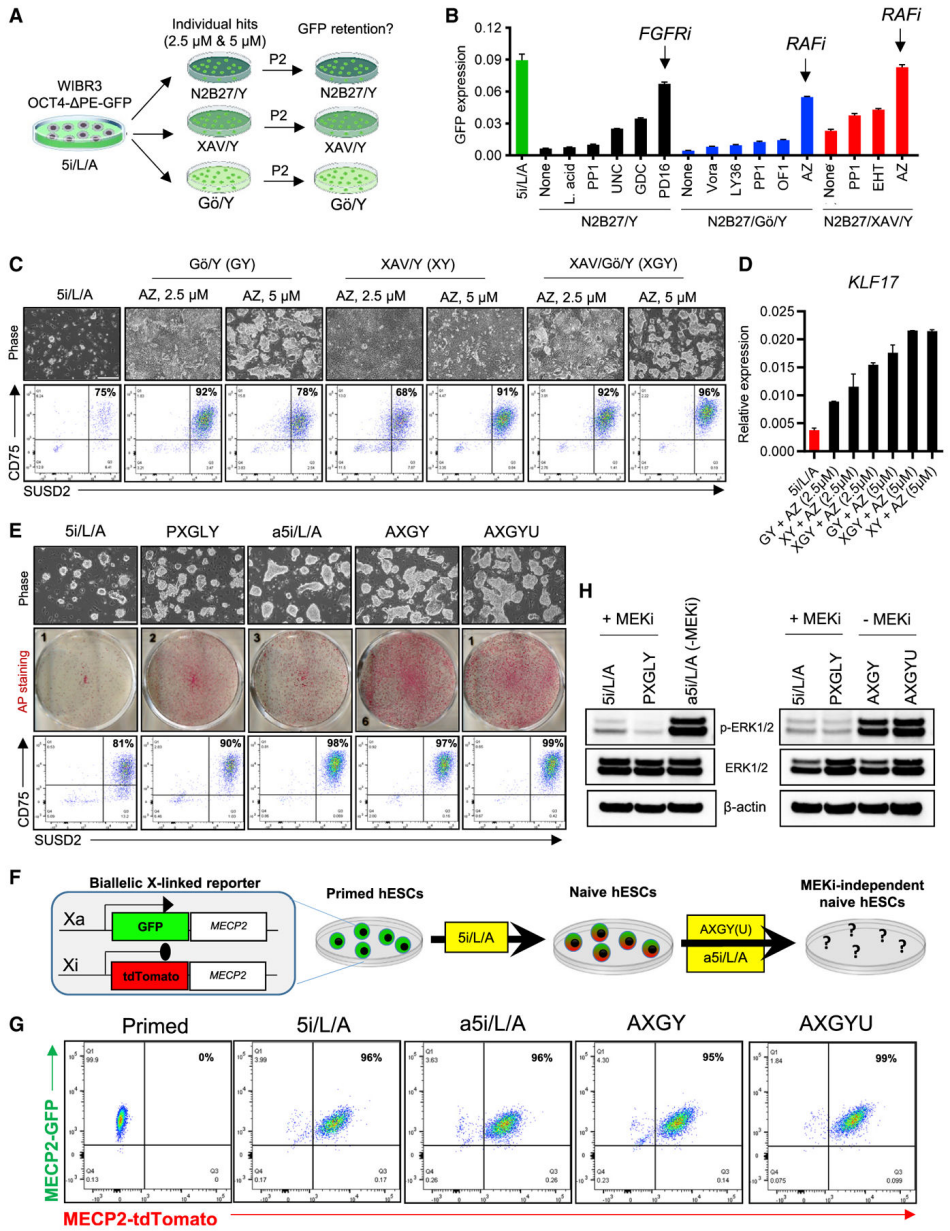
Defining the activity of selected hit compounds during extended maintenance assays for naive human pluripotency (A) Experimental scheme for extended maintenance assays to evaluate the efficacy of hit compounds using WIBR3 OCT4-ΔPE-GFP^+^ naive hESCs derived in 5i/L/A. (B) Quantitative gene-expression analysis for *GFP* in naive hESCs that were switched from 5i/L/A to the indicated culture conditions for two passages. In this experiment, compounds were applied at 2.5 μM concentration. Error bars indicate mean ± SD of three technical replicates. Data are representative of two biological replicates. (C) Phase-contrast images (top) and flow-cytometry analyses using naive-specific CD75 and SUSD2 antibodies (bottom) in H9-naive hESCs that were switched from 5i/L/A to the indicated culture conditions for two passages. The scale bar depicts 250 μm. (D) Quantitative gene-expression analysis for the naive-specific transcription factor *KLF17* in samples shown in (C). Error bars indicate mean ± SD of three technical replicates. (E) Phase-contrast images (top), alkaline phosphatase (AP) staining (middle), and flow-cytometry analyses using naive-specific CD75 and SUSD2 antibodies (bottom) in H9-naive hESCs that were switched from 5i/L/A and maintained in four different naive conditions for two passages. Data are representative of two biological replicates. The scale bar depicts 250 μm. (F) Experimental scheme for evaluating the capacity of alternative naive maintenance conditions to maintain biallelic X-linked reporter activity using WIBR3 *MECP2-GFP/tdTomato* reporter hESCs. (G) Flow-cytometry analysis for GFP and tdTomato in naive WIBR3 *MECP2-GFP/tdTomato* reporter hESCs that were derived from the primed state in 5i/L/A and thereafter maintained in a5i/L/A, AXGY, or AXGYU for two passages. (H) Western blot analysis for p-ERK, total ERK, and β-actin in H9-naive hESCs derived from the primed state in 5i/L/A and switched to PXGLY (Bredenkamp et al., 2019b), a5i/L/A, AXGY, or AXGYU. See also Figure S2 and Table S3.

We investigated whether 13 hit compounds that could be readily procured from commercial vendors (Figure 1E) were capable of maintaining naive human pluripotency over multiple passages in the absence of 2i. For this purpose, we used WIBR3 OCT4-ΔPE-GFP^+^-naive hESCs that were generated in the absence of reprogramming transgenes using the 5i/L/A cocktail (Theunissen et al., 2014). Naive hESCs were seeded in 6-well plates and transferred from 5i/L/A to the 17 different 2i-independent media formulations identified in our high-throughput screens (3/13 hits showed activity in multiple screens and were therefore tested in multiple basal media) (Figure 1E; Table S3). Compounds were tested at two different concentrations (2.5 and 5 μM), and cells were maintained for two passages (Figure 2A). Imaging and quantitative reverse transcriptase PCR (qRT-PCR) analysis indicated that only 3 of these compounds showed significant activity in maintaining OCT4-ΔPE-GFP reporter activity after passaging: the ERK1/2 inhibitor GDC-0994 and the FGFR1 inhibitor PD166866 showed activity in the presence of ROCK inhibitor (Y) alone, while the pan-RAF inhibitor AZ628 showed activity in Gö/Y and XAV/Y (Figure 2B; Figures S2A and S2B).

We initially focused on the pan-RAF inhibitor AZ628, which supported robust expansion of OCT4-ΔPE-GFP^+^ cells in the presence of either XAV or Gö. We first asked whether naive hESC self-renewal could be enhanced by combining AZ628, XAV, Gö, and Y-27632 together in a single cocktail (AXGY). Indeed, this combination enabled homogeneous expansion of naive hESCs as measured by flow-cytometry analysis for the cell-surface markers CD75 and SUSD2, which are enriched in naive compared to primed hESCs (Bredenkamp et al., 2019a; Collier et al., 2017), and qRT-PCR analysis for the naive-specific transcription factor *KLF17*, which is expressed in the human blastocyst (Blakeley et al., 2015) (Figures 2C, 2D, and S2C). We then investigated whether the addition of the other hit compounds to the AXGY cocktail could further boost the expansion, purity, and/or gene expression of naive hESCs (Figures S2D and S2E). Notably, the SRC family kinase inhibitor Naphtol-PP1, which scored in all three of our high-throughput screens, enhanced dome-shaped colony morphology. A similar effect was seen with the SRC inhibitor WH-4-023, which is included in the 5i/L/A cocktail (Theunissen et al., 2014). However, SRC inhibition had little impact on expression of naive-specific cell-surface markers or transcript levels and was therefore not included for further analysis. The G9a/GLP inhibitor UNC0638 further stimulated *KLF17* expression, although colony proliferation was slightly reduced compared to AXGY (Figures S2D and S2E).

We also tested whether AZ628 could substitute for the MEK inhibitor PD03 in the 5i/L/A cocktail. This culture formulation, which we designated as alternative 5i/L/A (a5i/L/A), maintained a high level of OCT4-ΔPE-GFP activity (Figures S2F-S2H) and enhanced the purity of CD75/SUSD2 double-positive cells compared to 5i/L/A in both wild-type H9 and WIBR3 cells (Figure 2E; Figure S2I). However, naive hESCs maintained in AXGY displayed markedly enhanced colony formation efficiency compared to 5i/L/A or a5i/L/A (Figure 2E; Figure S2I). This suggests that the combination of TNKS and PKC inhibitors provides a signaling milieu that is more conducive for naive cell expansion. Colony formation efficiency in AXGY was more comparable to PXGLY, which is a modified version of the t2i/L/Gö cocktail in which GSK3 inhibition is replaced by TNKS inhibition (Bredenkamp et al., 2019a, 2019b) (Figure 2E). We also examined whether these alternative naive conditions can maintain biallelic expression of a dual X-linked fluorescent reporter line, which is activated upon primed-to-naive resetting in 5i/L/A (An et al., 2020; Theunissen et al., 2016) (Figure 2F). Indeed, a5i/L/A, AXGY, and AXGY supplemented with the G9a/GLP inhibitor UNC0638 (AXGYU) were all capable of maintaining MECP2-GFP/tdTomato double-positive cells over two passages, suggesting that both X chromosomes remain active under these alternative naive conditions (Figure 2G).

AZ628 is a pan-RAF inhibitor for BRAF and CRAF (RAF1) (Wenglowsky et al., 2012). Since the RAF kinases are located upstream of MEK1/2 in the MAPK signaling cascade, we expected that RAF inhibition should phenocopy MEK inhibition and suppress the downstream phosphorylation of ERK1/2. Surprisingly, switching naive hESCs from 5i/L/A to the alternative naive maintenance media resulted in stimulation of phosphorylated (p)-ERK levels (Figure 2H). This result indicates that the complete suppression of ERK phosphorylation is dispensable for maintenance of several hallmarks of naive human pluripotency, such as robust expression of *KLF17*, OCT4-ΔPE-GFP reporter activity, and biallelic *MECP2* expression. It also suggests that the pan-RAF inhibitor AZ628 may stimulate naive human pluripotency through mechanisms that are located upstream of ERK, such as blockade of other RAF or MEK targets. Alternatively, the effect of AZ628 could be mediated through inhibition of lower affinity kinase targets, which include Epha2, PDGFRA, p38 alpha, LCK, and RET (Wenglowsky et al., 2012).

### Naive hESCs maintained with a pan-RAF inhibitor retain a pre-implantation identity

**Figure 3.**
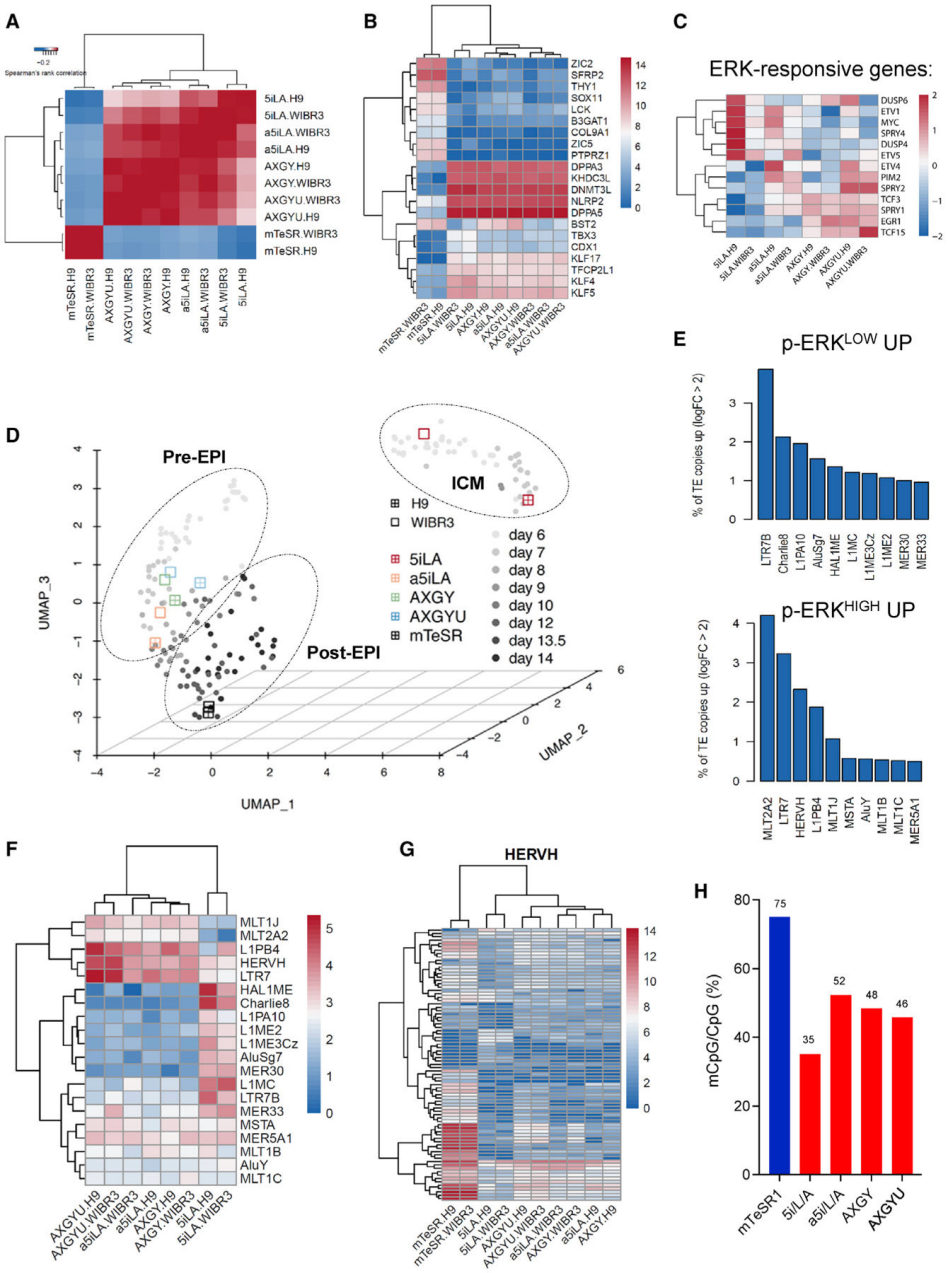
Molecular profiling of alternative naive hESCs maintained in the absence of 2i (A) Heatmap of RNA-seq data from primed hESCs maintained in mTeSR1 and naive hESCs that were derived in 5i/L/A and subsequently maintained in 5i/L/A or three alternative naive media (a5i/L/A, AXGY, or AXGYU) for two passages. Hierarchical clustering of Spearman’s rank correlation coefficients between samples was performed considering significantly differentially expressed genes (DEGs) (abs(log2FC) >1.5, adj p < 0.05). Data are shown for two independent genetic backgrounds (H9 and WIBR3 hESC lines). (B) Expression heatmap of selected naive and primed-specific markers in the samples described in (A). (C) Expression heatmap of selected ERK-responsive target genes in the samples described in (A). (D) UMAP dimension reduction analysis of single cell RNA-seq data representing ICM, Pre-EPI, and Post-EPI from 3D-cultured human embryos (Xiang et al., 2020) compared to the naive and primed samples described in (A). Clusters are drawn to indicate *in vivo* samples as shown in Xiang et al. (2020). (E) TE families upregulated in 5i/L/A (p-ERK^LOW^ UP) versus three alternative naive maintenance conditions (p-ERK^HIGH^ UP). Histograms indicate the percentages of TE copies that are upregulated by logFC >2 in either sample group. (F) Heatmap indicating differentially expressed TE families between naive H9 and WIBR3 hESCs maintained in 5i/L/A or three alternative naive maintenance conditions. (G) Heatmap indicating expression of individual HERVH integrants in primed H9 and WIBR3 hESCs and naive hESCs maintained in 5i/L/A or three alternative naive maintenance conditions. (H) Genome-wide CpG methylation level of all H9 samples described in (A) based on WGBS five passages after switching from 5i/L/A to the alternative naive media. An accompanying tile-based measure of global DNA methylation is shown in Figure S3G. See also Figure S3.

To define the transcriptional identity of naive hESCs maintained with AZ628, we performed RNA-seq analysis on H9 and WIBR3-naive hESCs that were derived in 5i/L/A and subsequently transferred to a5i/L/A, AXGY, or AXGYU. Hierarchical clustering based on significantly differentially expressed genes (DEGs) perfectly separated naive and primed hESCs (Figure 3A; Table S4). Within the naive branch, the alternative naive media formed a separate cluster from the 5i/L/A samples. Overall, all naive conditions exhibited highly similar expression of typical naive-specific transcription factors, such as *DPPA3, DPPA5, DNMT3L, NLRP2*, and *KHDC3L* (Figure 3B). Volcano-plot analyses indicated consistent upregulation of the ERK-responsive negative feedback regulator *SPRY1* and the transcription factor *GLI2* in the alternative naive conditions, while the 5-methylcytosine hydroxylase *TET2* and markers associated with extraembryonic lineages (e.g., *GATA6, GCM15, HAND1, KRT18, TFAP2A*) were upregulated in 5i/L/A (Figures S3A-S3C). The alternative naive hESCs that were maintained without MEK1/2 inhibitors also showed upregulation of the ERK-responsive genes *ERG1, SPRY2*, and *TCF3* (Figure 3C).

Comparison to scRNA-seq analysis of 3D-cultured human embryos (Xiang et al., 2020) segregated 5i/L/A and the alternative naive conditions into two distinct clusters: while 5i/L/A samples were more closely aligned with the inner cell mass (ICM) at days 6–7 of human development, naive hESCs maintained in AXGY, AXGYU, or a5i/L/A clustered more closely with pre-implantation epiblast (EPI) cells at days 7–8 of development (Figure 3D). In contrast, primed hESCs in mTeSR media aligned more closely with post-implantation EPI cells at days 12–14 of development, which is consistent with a prior comparison to non-human primate embryos (Nakamura et al., 2016). These data indicate that naive hESCs maintained with the pan-RAF inhibitor AZ628 reside in a more advanced stage of human EPI development yet retain expression of typical marker genes associated with naive human pluripotency.

Since naive and primed hESCs can be distinguished by expression of developmentally programmed TE families (Pontis et al., 2019; Theunissen et al., 2016), we also examined the transposon transcription profile or “transposcriptome” under alternative naive conditions. Naive cells in 5i/L/A showed elevated expression of the LTR7B TE subfamily, while transfer to AXGY, AXGYU, or a5i/L/A resulted in a moderate increase in expression of LTR7 and HERVH integrants (Figures 3E-3G and S3D), which were previously shown to be upregulated in primed hESCs (Theunissen et al., 2016). This effect was most pronounced upon addition of the G9a/GLP inhibitor UNC0638. However, the activation of LTR7 and HERVH elements was far more extensive and significant in primed hESCs, which indicates that LTR7/HERVH expression is dynamic across different stages of EPI development captured *in vitro*. In contrast, expression of naive-enriched SVA-D and HERVK integrants was largely invariable between 5i/L/A and the alternative naive conditions (Figures S3E and S3F). These data provide a further confirmation that full suppression of ERK phosphorylation is dispensable for maintaining key hallmarks of naive human pluripotency.

Globally reduced DNA methylation levels are an intrinsic feature of mammalian pre-implantation development that is recapitulated in naive stem cell culture (Hackett et al., 2013; Lee et al., 2014; Leitch et al., 2013; von Meyenn et al., 2016). Studies in the mouse system have suggested that MEK1/2 inhibition induces global hypomethylation via impairment of DNA methylation enzymes (Choi et al., 2017; Yagi et al., 2017). We examined DNA methylation levels under alternative naive conditions by whole-genome bisulfite sequencing (WGBS). Overall CpG DNA methylation levels increased from ~35% in 5i/L/A naive hESCs to ~45% in AXGY(U) and >50% in a5i/L/A. These DNA methylation levels are slightly elevated compared to the level of DNA methylation reported in the human ICM (~42%) (Guo et al., 2014) but remain significantly lower compared to the hypermethylated DNA signature in primed hESCs (~75%) (Figures 3H and S3G). Despite the overall increase in DNA methylation, increased expression of HERVH integrants in alternative naive conditions was correlated with locally reduced methylation levels (Figure S3H). In addition, DNA methylation at imprinted DMRs was depleted under all examined conditions (Figure S3I). This may be explained by the fact that these maintenance experiments were performed in naive hESCs that were derived from the primed state in 5i/L/A, which is known to cause imprint erasure within four passages (Theunissen et al., 2016).

### Inhibition of other enzymes in the FGFR-RAF-MEK-ERK pathway also sustains naive human pluripotency

**Figure 4.**
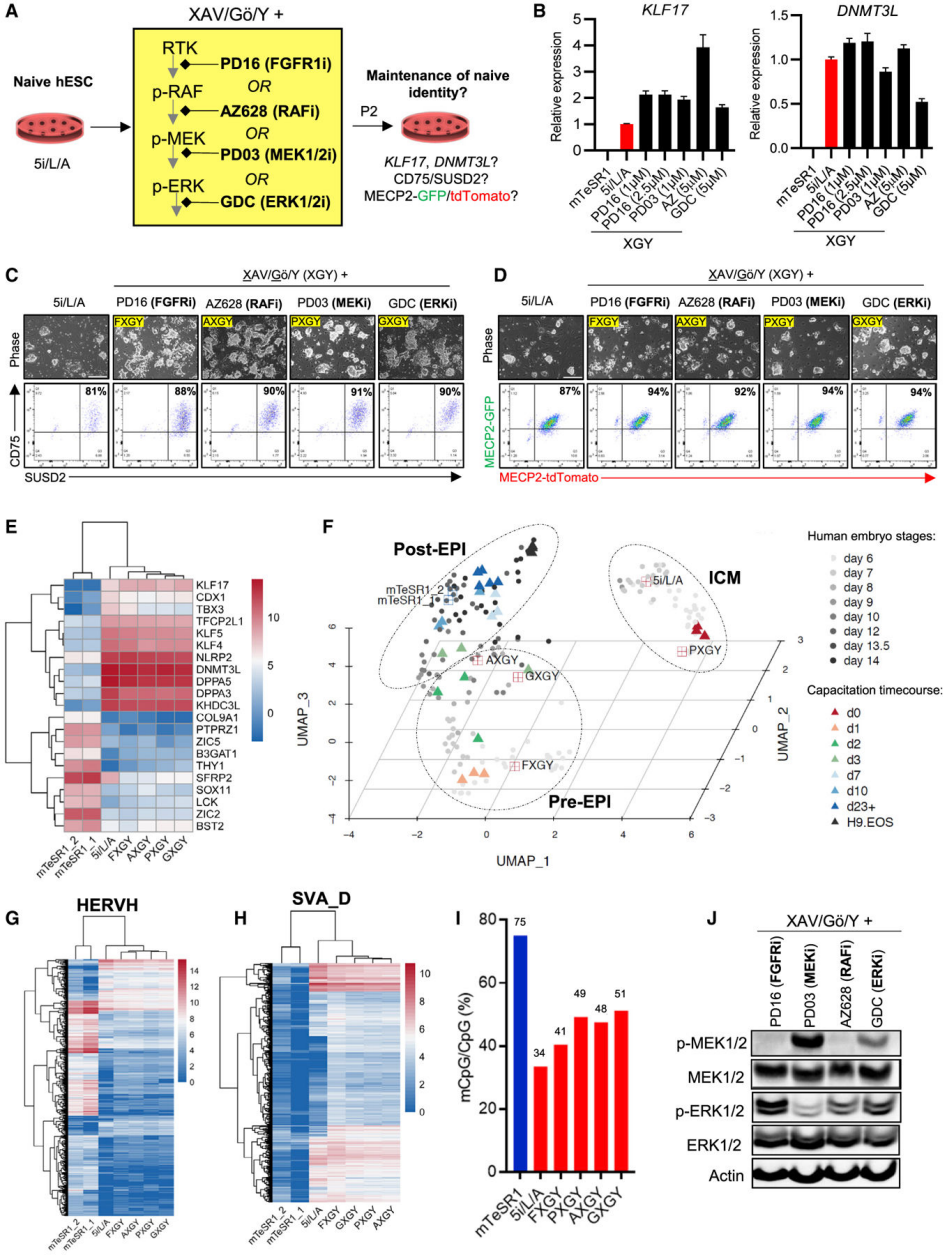
Inhibition of other enzymes in the FGFR-RAF-MEK-ERK pathway also maintains naive human pluripotency (A) Experimental scheme for extended maintenance assays to evaluate the efficacy of FGFR1, RAF, MEK, and ERK inhibitors in the presence of TNKS, PKC, and ROCK inhibition (XAV/Gö/Y). (B) Quantitative gene-expression analysis for the naive-specific transcripts *KLF17* and *DNMT3L* in the titration experiment shown in Figure S4C. PXGY (PD03-XGY) and AXGY (AZ628-XGY) are included as controls. Error bars indicate mean ± SD of three technical replicates. (C) Phase-contrast images (top) and flow-cytometry analyses using naive-specific CD75 and SUSD2 antibodies (bottom) in H9-naive hESCs that were switched from 5i/L/A to the four alternative naive maintenance media for two additional passages. Data are representative of two biological replicates. The scale bar depicts 250 μm. (D) Phase-contrast images (top) and flow-cytometry analyses (bottom) of naive WIBR3 *MECP2-GFP/tdTomato* reporter hESCs that were derived from the primed state in 5i/L/A and thereafter maintained in the four alternative naive maintenance media for two passages. The scale bar depicts 250 μm. (E) Expression heatmap of selected naive and primed-specific markers in H9 hESCs for the alternative naive maintenance conditions described in (C). Gene expression was compared to H9 mTeSR1 and 5i/L/A samples previously analyzed in Figure 3 and an additional H9 mTeSR1 sample (mTeSR1_2). (F) UMAP dimension reduction analysis of scRNA-seq data representing the ICM, Pre-EPI, and Post-EPI from 3D-cultured human embryos (Xiang et al., 2020) compared to the naive and primed samples described in (E). Clusters are drawn to indicate *in vivo* samples as shown in Xiang et al. (2020). A time-course RNA-seq analysis of naive hESCs undergoing capacitation into a formative pluripotent state (Rostovskaya et al., 2019) was also integrated into this UMAP. (G) Heatmap indicating expression of individual HERVH integrants in primed H9 hESCs and naive hESCs maintained in 5i/L/A or four alternative naive maintenance conditions as described in (E). (H) Heatmap indicating expression of individual SVA_D integrants in H9 primed hESCs and naive hESCs maintained in 5i/L/A or four alternative naive conditions as described in (E). (I) Genome-wide CpG methylation level of all H9 samples described in (C) based on whole-genome bisulfite sequencing five passages after switching from 5i/L/A to the four alternative naive maintenance conditions. Data were compared to the H9 mTeSR1 sample previously analyzed in Figure 3H. (J) Western blot analysis for p-MEK1/2, total MEK1/2, p-ERK1/2, total ERK1/2, and β-actin (loading control) protein levels in H9-naive hESCs derived from the primed state in 5i/L/A and switched to four alternative naive conditions. See also Figure S4 and Table S3.

The above results indicate that the pan-RAF inhibitor AZ628 can maintain key molecular features of naive human pluripotency in combination with TNKS, PKC, and ROCK inhibitors (XGY). Since AXGY promoted more robust expansion of naive cells compared to 5i/L/A, we asked whether any of the other hit compounds from our high-throughput screens could also sustain naive human pluripotency under these conditions. Naive hESCs were derived from the primed state in 5i/L/A and switched to serum-free media supplemented with XGY and the commercially available hit compounds (Tables S2 and S3). We also included 9 additional compounds that have a shared target annotation as the remaining hit compounds from our screens for which commercial vendors were unavailable (Figure S4A). In addition to AZ628, the only compounds that robustly sustained CD75/SUSD2-positive cells over multiple passages were the FGFR inhibitor PD166866 and the ERK inhibitor GDC-0994. While the MAPK14 (p38) inhibitor Semapimod supported some double-positive cells, these cells displayed very limited proliferation (Figure S4B). Thus, the only hit compounds that could efficiently replace MEK1/2 inhibitors during the long-term maintenance of naive human pluripotency inhibit either upstream (FGFR, RAF) or downstream (ERK1/2) kinases (Figure 4A). Titration experiments revealed that 1 μM of FGFR inhibitor was sufficient to maintain expression of naive-specific transcripts, while the ERK inhibitor was more effective at 5 μM Figure 4B; Figure S4C). We confirmed by flow cytometry that these inhibitors not only maintained homogeneous CD75/SUSD2 expression but also biallelic X-linked MECP2 fluorescent reporter activity (Figures 4C and 4D).

We then sought to determine whether naive hPSCs maintained with the FGFR inhibitor PD166866 (FXGY) or ERK inhibitor GDC-0994 (GXGY) reside in a similar state as those maintained in AXGY. Retention of canonical naive markers in the alternative naive maintenance conditions was corroborated by RNA-seq analysis, which also showed negligible expression of primed markers (Figures 4E and 4F; Table S4). Transfer from 5i/L/A to AXGY, FXGY, or GXGY captured the cells in a pre-implantation EPI identity, while continuous treatment with a MEK inhibitor (PXGY) drove them toward an ICM identity (Figure 4F). Similar results were obtained by comparison to cynomolgus macaque embryo stages (Nakamura et al., 2016), although the GXGY condition clustered more closely with macaque ICM (Figure S4D). AXGY, GXGY, and FXGY displayed a modest increase in HERVH integrants, while all naive maintenance conditions showed strong activation of SVA-D integrants (Figures 4G and 4H). Global DNA methylation was slightly elevated in all four XGY-based naive media compared to 5i/L/A but remained substantially reduced compared to the primed state (Figure 4I; Figure S4E). Furthermore, western blotting revealed increased p-ERK levels in AXGY, GXGY, and FXGY relative to PXGY (Figure 4J). Notably, GDC-0994 is known not to alter the phosphorylation of cellular ERK1/2 (Basken et al., 2018; Pegram et al., 2019). We conclude that inhibition of FGFR, RAF, MEK, or ERK can sustain bona fide features of naive human pluripotency despite variable levels of ERK phosphorylation. However, the only conditions that were capable of maintaining naive hESCs in a human ICM-like state were those that included a direct MEK inhibitor (5i/L/A and PXGY).

While naive hPSCs are not directly responsive to embryonic lineage inductive cues, they can be re-adapted to primed culture conditions (a process called “re-priming”) (Sahakyan et al., 2017; Theunissen et al., 2016) or transitioned into a lineage-competent formative state upon treatment with the TNKS inhibitor XAV939 (Rostovskaya et al., 2019). Naive hESCs derived in 5i/L/A and switched to three alternative maintenance media (AXGY, GXGY, or FXGY) acquired a primed morphology and activated the primed-specific cell-surface marker CD90 upon treatment with mTeSR1 media within two passages (Figures S4F and S4G). Furthermore, they downregulated naive-specific transcripts and activated formative markers during a 10-day capacitation experiment (Figure S4H). Consistent with their more advanced identity relative to human EPI development and a previously published capacitation time course (Figure 4F), naive hESCs maintained in AXGY showed more robust induction of formative markers compared to 5i/L/A (Figure S4H). Hence, naive hESCs maintained in the absence of a direct MEK inhibitor remain competent to re-enter the primed pluripotent state.

Since long-term culture in 5i/L/A has been associated with genomic instability (Di Stefano et al., 2018; Pastor et al., 2016; Theunissen et al., 2014), we performed karyotyping on naive cells that were derived in 5i/L/A and switched to alternative naive maintenance conditions. Naive hESCs that were continuously maintained in 5i/L/A contained various chromosomal rearrangements by passage 10. In contrast, a normal karyotype was maintained in naive hESCs that were switched from 5i/L/A to AXGY or FXGY while a small subset of abnormal cells was observed in either PXGY or GXGY (Figure S4I). Hence, transfer to XGY-based naive maintenance media may enhance the genomic stability of naive hESCs, although subclonal aneuplodies were still observed in some of the alternative maintenance conditions. We also verified that these cells maintained homogeneous expression of CD75 and SUSD2, indicating that naive hESCs can be maintained in the absence of a direct MEK inhibitor during extended culture (Figure S4J).

### Dual MEK and ERK inhibition promotes efficient primed-to-naive resetting in combination with activin A

**Figure 5.**
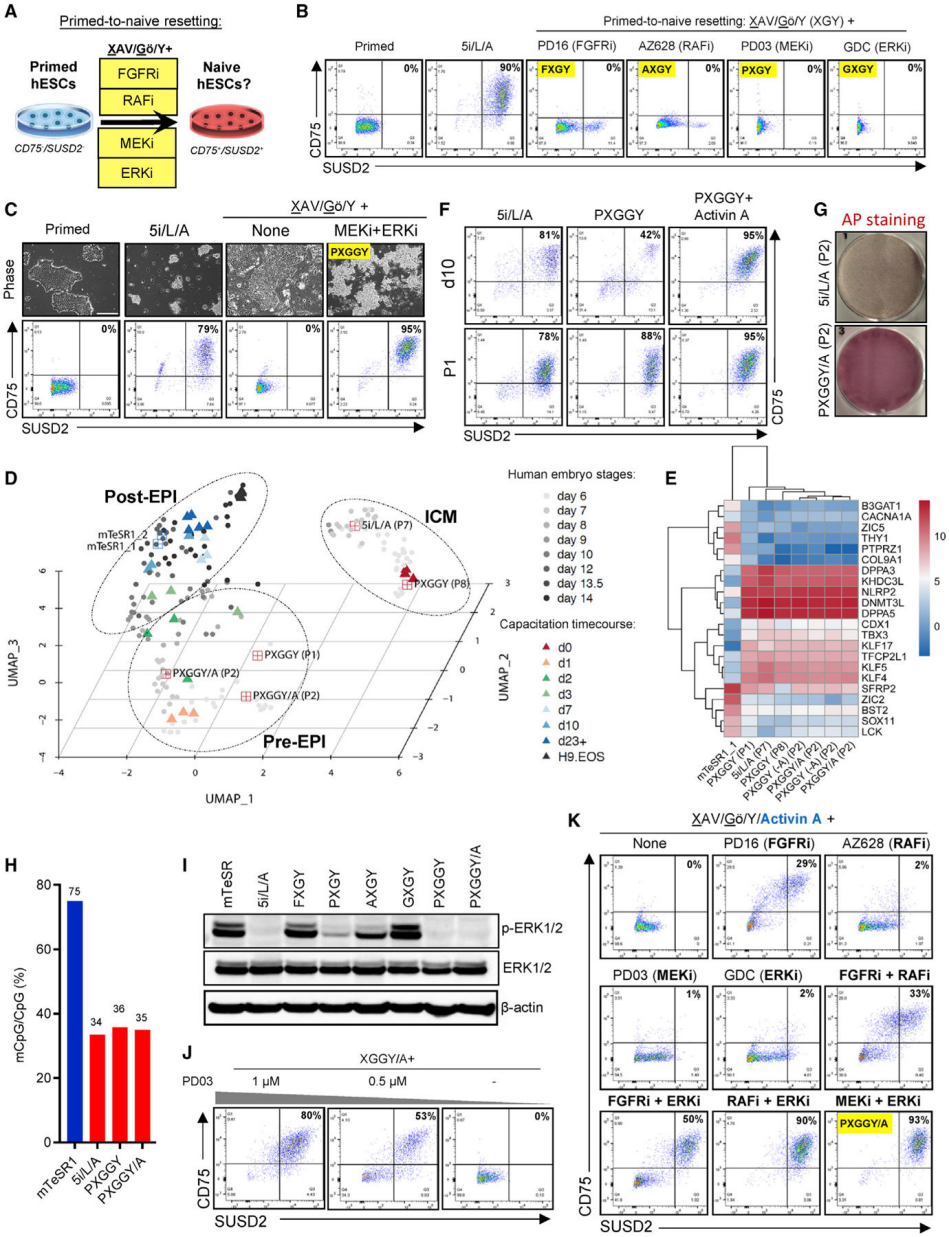
Examining the signaling requirements for primed-to-naive resetting (A) Experimental scheme for evaluating the efficacy of alternative naive maintenance media to induce naive pluripotency in primed hESCs. Successful induction of naive pluripotency was assessed by flow cytometry for the naive-specific cell-surface markers CD75 and SUSD2 at the end of P1. (B) Flow-cytometry analyses for naive-specific cell-surface markers CD75 and SUSD2 at the end of P1 of primed-to-naive conversion using H9 hESCs in 5i/L/A and four alternative naive maintenance conditions. Data are representative of two biological replicates. (C) Phase-contrast images (top) and flow-cytometry analysis for naive-specific cell-surface markers CD75 and SUSD2 (bottom) in H9 primed hESCs upon treatment with XAV939, Gö6983, and Y-27632 (XAV/Gö/Y) together with the MEK inhibitor PD0325901 and the ERK inhibitor GDC-0994 (PXGGY) at the end of P1. Data are representative of two biological replicates. The scale bar depicts 250 μm. (D) UMAP dimension reduction analysis of single cell RNA-seq data representing the ICM, Pre-EPI, and Post-EPI from 3D-cultured human embryos (Xiang et al., 2020) compared to naive hESCs that were derived in PXGGY at P1 and P8 or PXGGY+activin A(PXGGY/A) at P2. Expression data were compared to H9 mTeSR1 and 5i/L/A samples previously analyzed in Figure 3. Clusters are drawn to indicate *in vivo* samples as shown in (Xiang et al., 2020). A time-course RNA-seq analysis of naive hESCs undergoing capacitation into a formative pluripotent state (Rostovskaya et al., 2019) was also integrated into this UMAP. (E) Expression heatmap of selected naive and primed-specific markers in H9 hESCs for the primed-to-naive conversion conditions described in Figure 5C and Figure 5F. Naive hESCs derived in PXGGY were examined at the end of P1 and P8. We also examined naive hESCs that were derived in PXGGY/A and subsequently maintained in the presence or absence of activin A for two passages (PXGGY-A) in two biological replicates each. Expression data were compared to H9 mTeSR1 and 5i/L/A samples previously analyzed in Figure 3. (F) Flow-cytometry analysis using antibodies for the naive-specific cell-surface markers CD75 and SUSD2 in H9 primed hESCs upon treatment with 5i/L/A, PXGGY, and PXGGY/A at day 10 (top) and at the end of P1 (bottom) of primed-to-naive resetting. Data are representative of two biological replicates. (G) Alkaline phosphatase (AP) staining of H9 hESCs after two passages of primed-to-naive conversion in 5i/L/A and PXGGY/A. Data are representative of two biological replicates. (H) Genome-wide CpG DNA methylation level of H9-naive hESCs that were converted in 5i/L/A or PXGGY/A at P5. We also examined naive hESCs that were derived in PXGGY/A and maintained for four additional passages in the absence of activin A. Data were compared to the H9 mTeSR1 and 5i/L/A samples previously analyzed in Figure 4I. (I) Western blot analysis for p-ERK1/2, total ERK1/2, and β-actin (loading control) protein levels in H9 primed hESCs treated with indicated media. (J) Flow-cytometry analysis using antibodies for the naive-specific cell-surface markers CD75 and SUSD2 to assess the effect of MEK inhibitor PD0325901 titration during primed-to-naive resetting of H9 primed hESCs in PXGGY/A medium at the end of P1. (K) Flow-cytometry analysis using antibodies for the naive-specific cell-surface markers CD75 and SUSD2 during primed-to-naive resetting of H9 primed hESCs by various combinations of FGF pathway inhibitors at the end of P1. Data are representative of two biological replicates. See also Figure S5 and Table S3.

Finally, we examined whether the alternative naive maintenance formulations identified by our screens are also capable of inducing naive pluripotency in primed hESCs (Figure 5A; Figure S5A). H9 primed hESCs were seeded on MEFs and treated with 5i/L/A or alternative media and the expression of naive-specific cell-surface markers was examined by flow cytometry. Remarkably, CD75/SUSD2 double-positive cells were observed only upon treatment with 5i/L/A (Figure 5B; Figure S5B). This suggests that the use of a MEK inhibitor in the 5i/L/A cocktail is critical for inducing naive pluripotency, but neither MEK nor ERK inhibition is sufficient to induce CD75/SUSD2 double-positive cells in combination with TNKS, PKC, and ROCK inhibitors (XGY). This led us to investigate whether the use of multiple FGF pathway inhibitors might facilitate primed-to-naive resetting together with XGY. While some CD75/SUSD2 double-positive cells were observed upon dual inhibition of RAF and either MEK or ERK, these conditions were cytotoxic (data not shown). In contrast, more robust induction of double-positive cells was observed upon dual inhibition of MEK and ERK (Figure 5C). We refer to this naive induction cocktail as PXGGY for PD0325901 (MEKi), XAV939 (TNKSi), Gö6983 (PKCi), GDC-0994 (ERKi), and Y-27632 (ROCKi) (Table S3).

We proceeded to further characterize naive hESCs derived in PXGGY. While these cells lacked the defined colony morphology observed in 5i/L/A, they acquired a pre-implantation EPI identity within one passage and further passaging resulted in transition toward an ICM-like state (Figures 5D and 5E; Table S4). They also maintained expression of naive-specific cell-surface markers during extended passaging (Figure S5C) and displayed a normal karyotype at P14 (Figure S5D). We also confirmed that PXGGY induced biallelic MECP2 reporter activity (Figure S5E). However, the efficiency of CD75/SUSD2 double-positive cells within the first 10 days of conversion remained low, leading us to examine whether provision of additional cytokines might facilitate the primed-to-naive transition. An intriguing candidate is recombinant activin A, which was included in the 5i/L/A cocktail in order to enhance cell survival during primed-to-naive resetting (Theunissen et al., 2014). Indeed, addition of activin A to PXGGY (PXGGY/A) enhanced naive conversion efficiency, resulting in accelerated reprogramming kinetics as measured by flow cytometry on day 10 and colony formation efficiency at P2 (Figures 5F and 5G). Naive hESCs derived in PXGGY/A acquired a pre-EPI identity within two passages (Figure 5D) and activin A could be withdrawn (PXGGY-A) without adversely affecting the expression of key naive markers (Figure 5E). Naive hESCs derived in PXGGY/A underwent a similar global reduction in DNA methylation and imprint erasure as those derived in 5i/L/A, which is likely attributable to the inclusion of a direct MEK inhibitor (Figure 5H; Figure S5F).

Recent studies from our laboratory and others have shown that naive hPSCs have an enhanced potential for extraembryonic differentiation and can give rise to human trophoblast stem cells (hTSCs) (Castel et al., 2020; Cinkornpumin et al., 2020; Dong et al., 2020; Guo et al., 2021; Io et al., 2021). Naive hESCs that were derived in PXGGY/A and maintained without activin A for two passages acquired a typical hTSC-like morphology and displayed activation of the hTSC-specific cell-surface markers EGFR and ITGA6 upon treatment with hTSC media (Okae et al., 2018) (Figures S5G and S5H). They also upregulated the primed-specific cell-surface marker CD90 upon re-priming in mTeSR1 media (Figures S5G and S5I). Hence, naive hESCs derived in PXGGY/A respond in comparable manner to trophoblast and re-priming conditions as those derived in 5i/L/A. We also confirmed that naive hESCs derived in PXGGY/A could be switched to the three alternative MEKi-independent maintenance media (AXGY, GXGY, and FXGY), while sustaining the expression of naive-specific cell-surface markers (Figures S5J and S5K) and their trophoblast potential (Figures S5J and S5L).

Treatment of primed hESCs with the naive induction cocktails 5i/L/A, PXGGY, or PXGGY/A strongly reduced p-ERK levels within 24 h, while p-ERK levels were maintained or only partially reduced upon treatment with alternative naive maintenance media that failed to induce CD75/SUSD2 double-positive cells (Figure 5I). These results suggest that the use of a direct MEK inhibitor is necessary, but not sufficient, to achieve full suppression of pERKin primed hESCs and facilitate the transition to naive pluripotency. Consistent with this interpretation, titration or removal of the MEK inhibitor rapidly compromised reprogramming efficiency using the PXGGY/A cocktail (Figure 5J). However, we considered whether it might be possible to bypass the use of a direct MEK inhibitor during primed-to-naive resetting by combining other FGF pathway inhibitors with activin A. Indeed, heterogeneous induction of some CD75/SUSD2 double-positive cells was observed by treating primed hESCs with the FGFR inhibitor PD166866 in the presence of XGY and activin A (Figure 5K). Furthermore, several combinations of FGF pathway inhibitors enabled more robust induction of naive cells, including FGFRi+RAFi, FGFRi+ERKi, and RAFi+ERKi (Figure 5K). These combinations were also able to induce MECP2-GFP/tdTomato double-positive cells, although conversion kinetics were not as efficient as in PXGGY/A (Figure S5M). Hence, the use of a direct MEK inhibitor does not appear to be absolutely required for primed-to-naive resetting but can be circumvented by combining other FGF pathway inhibitors in an optimized signaling environment (i.e., containing TNKS, PKC, ROCK inhibitors, and recombinant activin A).

## DISCUSSION

The past decade has witnessed substantial interest in the isolation of naive hPSCs that correspond to pluripotent cells in the human pre-implantation embryo. Significant progress has been made toward capturing bona fide naive hPSCs by primed-to-naive resetting (Guo et al., 2017; Takashima et al., 2014; Theunissen et al., 2014), deriving naive hESCs directly from isolated ICM cells (Guo et al., 2016), and reprogramming somatic cells to pluripotency under naive conditions (Bredenkamp et al., 2019b; Giulitti et al., 2019; Kilens et al., 2018; Liu et al., 2017; Wang et al., 2018). However, a detailed understanding of the signaling requirements for inducing and maintaining naive human pluripotency has remained elusive. In an effort to expand the known repertoire of factors regulating naive human pluripotency, we performed high-throughput chemical screening using a library of ~3,000 well-annotated compounds (Canham et al., 2020) to identify alternative compounds that can maintain naive hESCs in the absence of MEK and GSK3 inhibitors that are commonly included in naive stem cell protocols.

**Figure 6.**
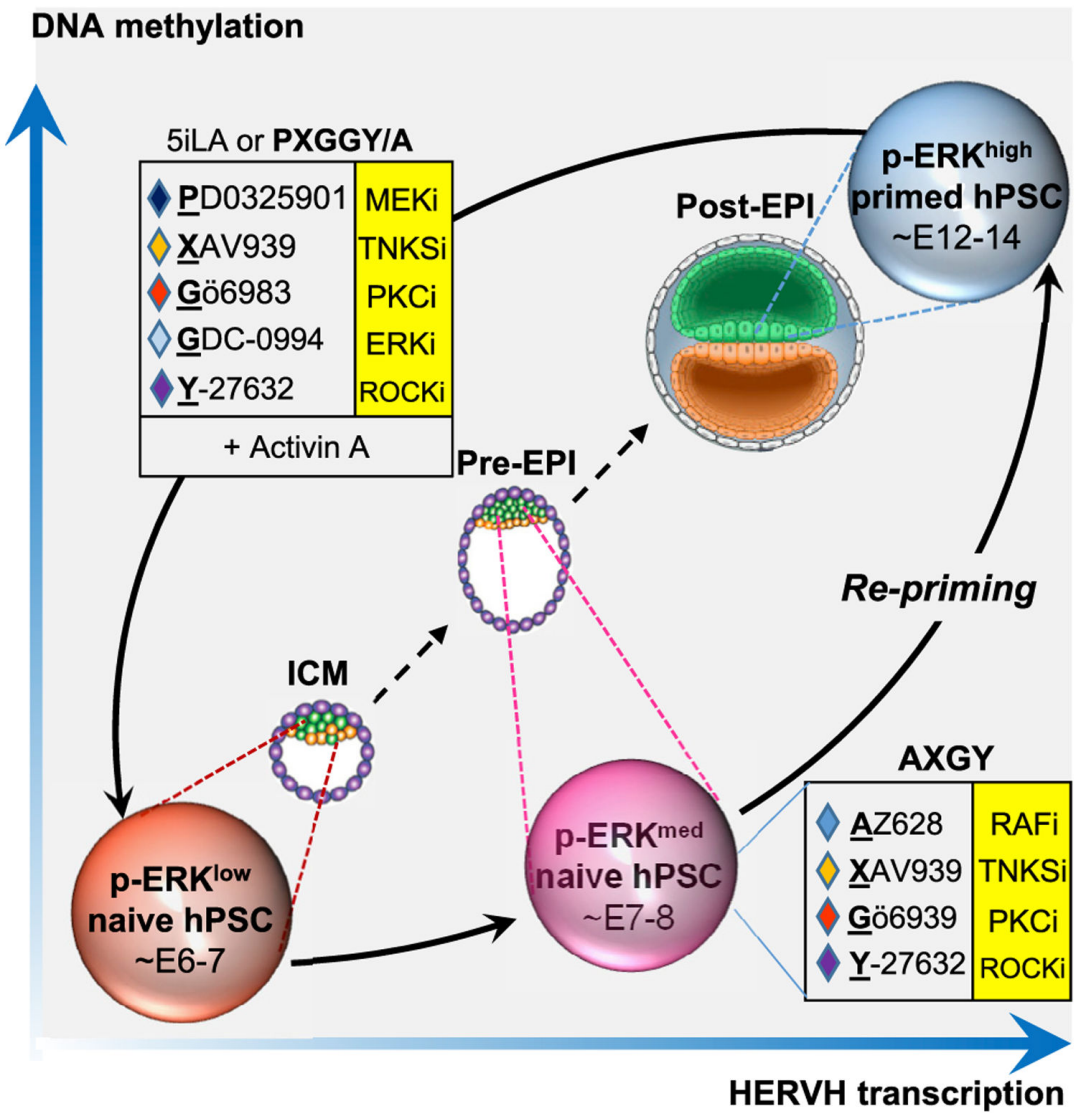
Distinct signaling requirements for inducing and maintaining naive human pluripotency Model summarizing the main findings from this study: dual MEK and ERK inhibition promotes efficient primed-to-naive resetting in combination with TNKS, PKC, ROCK inhibitors, and activin A (PXGGY/A). These p-ERK^LOW^ naive hESCs resemble previously described 5i/L/A naive hESCs (Theunissen et al., 2016; Theunissen et al., 2014) and exhibit a human ICM-like identity based on comparison to 3D-cultured human embryos (Xiang et al., 2020). They can transition into a pre-implantation EPI state with elevated levels of ERK phosphorylation in the presence of RAF, TNKS, PKC, and ROCK inhibitors (AXGY). These cells retain expression of typical naive markers but also display increased levels of global DNA methylation and HERVH transcription. Global DNA methylation and HERVH transcription are further increased in the primed pluripotent state, which corresponds to the post-implantation epiblast at E12–14.

Our results demonstrate that MEK inhibitors can be replaced by inhibitors of both upstream (FGFR1, RAF) and downstream (ERK) kinases during the maintenance of naive human pluripotency. The most robust expansion of naive hESCs was attained in combination with TNKS, PKC, and ROCK inhibitors (XGY), in agreement with recent work from the Smith laboratory (Bredenkamp et al., 2019a, 2019b). Naive hESCs maintained by FGFR, RAF, or ERK inhibitors displayed multiple hallmarks of naive human pluripotency, including OCT4-ΔPE-GFP activity, biallelic X-linked reporter activity, and expression of key naive pluripotency genes. Surprisingly, ERK phosphorylation was stimulated in naive hESCs maintained with the RAF inhibitor AZ628 (AXGY) or FGFR inhibitor PD166866 (FXGY). Interestingly, however, modulation of these different nodes in the FGF pathway isolated naive hESCs along progressive stages of early development: while the inclusion of a direct MEK inhibitor in 5i/L/A or PXGY captured naive cells in a human ICM-like state, naive cells maintained in the absence of MEK inhibitors progressed to a pre-implantation EPI identity and displayed increased expression of HERVH integrants (Figure 6).

The alternative naive maintenance formulations were unable to induce naive pluripotency in primed hESCs, suggesting that complete MEK/ERK inhibition achieved by the 5i/L/A cocktail is critical for primed-to-naive resetting. Dual inhibition of MEK and ERK in the presence of PKC, TNKS, and ROCK inhibitors provided an alternative naive induction cocktail, which we refer to as PXGGY. When combined with activin A, this cocktail accelerated the activation of naive-specific cell-surface markers and biallelic X-linked reporter activity compared to 5i/L/A. However, naive hESCs generated with PXGGY/A still incurred imprint erasure, likely due to the inclusion of a direct MEK inhibitor (Choi et al., 2017; Yagi et al., 2017). In the presence of XGY and activin A naive cell induction could also be achieved by several other combinations of FGF pathway inhibitors, including FGFRi+RAFi, FGFRi+ERKi, and RAFi+ERKi, but reprogramming kinetics were reduced compared to PXGGY/A. This may provide a path to generate naive hESCs in the absence of direct MEK inhibition, although it remains to be determined whether these cells meet stringent criteria for naive pluripotency.

This work raises several questions for future investigation. First, it remains unclear how ERK phosphorylation is stimulated in the presence of upstream FGF pathway inhibitors. A potential mechanism involves the loss of negative feedback regulation (Lake et al., 2016), which could be explored by perturbing the expression of members of the DUSP and Sprouty families. Our results also raise the possibility that upstream FGF pathway inhibitors may stimulate naive human pluripotency through mechanisms that are located upstream of ERK, for example by blocking other RAF or MEK targets. A more complete understanding of the underlying biochemical mechanisms will likely require examination of the global phosphoproteome in naive hESCs maintained with different FGF pathway inhibitors. Second, the alternative naive maintenance conditions resulted in a progression in pre-implantation epiblast identity and increased HERVH expression compared to MEK inhibitor-containing naive media. This suggests that the alternative naive hESCs may be more responsive to embryonic lineage cues, while their enhanced proliferation may be beneficial for efforts to improve the contribution of naive hESCs to interspecies chimeras (Wu et al., 2016) or human blastocyst-like structures (Yu et al., 2021). Third, it will be important to evaluate which combination of naive induction and maintenance conditions best preserves the long-term genomic integrity of naive hESCs, while simultaneously mitigating the erosion of parent-specific DNA methylation marks at imprinted loci.

Finally, we wish to emphasize that we took a deliberately minimalistic approach by screening for factors that induce and maintain naive human pluripotency in the presence of few other signaling inputs. It may be possible to further improve on aspects of the naive phenotype by testing other permutations among the compounds identified in our screen or providing additional factors. Furthermore, our studies were performed on naive hESCs induced from pre-existing primed hESC lines. Whether similar signaling principles apply during naive hPSC derivation from blastocysts or somatic cells will require further examination. For example, it is conceivable that naive hPSCs may be isolated under less stringent conditions from pre-implantation embryos or reprogramming intermediates that already exist in a globally hypomethylated state. The high-throughput screens presented in this study expand the chemical toolkit available to capture hPSC states corresponding to discrete stages of embryogenesis.

## STAR★METHODS

### RESOURCE AVAILABILITY

#### Lead contact

Requests for further information should be directed to and will be fulfilled by the Lead Contact, Thorold Theunissen (t.theunissen@wustl.edu).

#### Materials availability

Requests for resources and reagents should be directed to and will be fulfilled by the Lead Contact, Thorold Theunissen (t.theunissen@wustl.edu).

#### Data and code availability

The RNA-seq and WGBS data are available under GEO: GSE153215.

### EXPERIMENTAL MODEL AND SUBJECT DETAILS

#### Cell lines and culture conditions

Primed hESCs (H9, WIBR3, WIBR3 *OCT4-ΔPE-GFP*, WIBR3 *MECP2-GFP/tdTomato*) were cultured in mTeSR Plus (STEMCELL Technologies, 100-0276) on Matrigel (Corning, 354277) coated wells and passaged using ReLeSR (STEMCELL Technologies, 05872) or dissociated with Dispase (STEMCELL Technologies, 07923) and passaged by cutting colonies into small, uniform squares with StemPro EZPassage Stem Cell Passaging Tool (GIBCO, 23181010) every 4 to 6 days. Primed hESCs were fed with fresh media every second day and were cultured in 5% CO_2_ and 20% O_2_ at 37°C. Naive hESCs were cultured in 5% CO_2_ and 5% O_2_ at 37°C in 5i/L/A or alternative culture conditions. 5i/L/A media were prepared by combining N2B27 and the following small molecules and cytokines as previously described (Theunissen et al., 2014): 1 μM PD0325901 (Stemgent, 04-0006), 1 μM IM-12 (Enzo, BML-WN102), 0.5 μM SB590885 (Tocris, 2650), 1 μM WH-4-023 (A Chemtek, H620061), 10 μM Y-27632 (Peprotech, 1293823), 20 ng/mL recombinant human LIF (PeproTech, 300-05) and 10 ng/mL Activin A (Peprotech, 120-14). 500 mL N2B27 was generated by combining: 240 mL DMEM/F12 (GIBCO, 11320), 240 mL Neurobasal (GIBCO, 21103), 5 mL N2 100X supplement (GIBCO, 17502), 10 mL B27 50X supplement (GIBCO, 17504), 1X GlutaMAX, 1X MEM NEAA (GIBCO, 11140), 0.1 mM β-mercaptoethanol (Millipore Sigma, 8.05740), 1% penicillin-streptomycin, and 50 μg/ml BSA Fraction V (GIBCO, 15260). All tissue culture experiments were performed in 6-well plates unless stated otherwise. Media were filtered using a 0.22 μm filter and cell lines were regularly tested for mycoplasma contamination. Detailed information on conditions used for defining alternative naive induction and maintenance media can be found in the Method details below and in Table S3.

### METHOD DETAILS

#### High-throughput chemical screening

For high-throughput screening, we used WIBR3 OCT4-ΔPE-GFP+ naive hESCs generated with inducible KLF2 and NANOG transgenes, as described previously (Theunissen et al., 2014). These cells were maintained in 1 μM PD0325901, 1 μM CHIR99021, and 2 μg/ml DOX (Sigma) (2i/DOX). For compound screening in presence of 2i/Y, 3,000 OCT4-ΔPE-GFP+ naive hESCs were seeded per well in vitronectin-coated 384 well plates in serum-free N2B27 media supplemented with 1 μM PD0325901, 1 μM CHIR99021, and 10 μM Y-27632. The next day individual compounds from the Novartis Mechanism of Action (MoA) library were applied at two concentrations (2.5 μM and 5 μM) in duplicate using an Echo 550 Acoustic Liquid Handler (Labcyte), and compounds were refreshed on day 4. Images were analyzed on day 5 post-DOX withdrawal using a Yokogawa Cell Voyager 7000 High-Content Imaging System in confocal mode using a 10x dry (NA = 0.45) objective lens using four fields per well that provided for full well coverage. While the screen was performed at two concentrations (2.5 μM and 5 μM), the lower concentration yielded few additional hits. Hit compounds were validated in 384 well plates across an 8-point concentration range in duplicates. For high-throughput screening in absence of 2i, 384 well plates were pre-coated with mitotically inactivated mouse embryonic fibroblasts (MEFs) to improve viability. Pilot assays revealed that a maximal Z’-factor was obtained in absence of 2i by performing image acquisition on day 7 post-DOX withdrawal and using a seeding density of 800 OCT4-ΔPE-GFP+ naive hESCs per 384 well. Naive hESCs were seeded in serum-free N2B27 media supplemented with 10 μM Y-27632 alone (N2B27/Y) or upon addition of 2 μM XAV939 (XAV/Y) or 2 μM Gö6983 (Gö/Y). The next day individual compounds from the Novartis MoA library were added at 5 μM concentration using the Echo 550 Acoustic Liquid Handler and compounds were refreshed on days 3 and 6. Images were acquired using the Yokogawa Cell Voyager 7000 on day 7 and analyzed as described above. Hit compounds were validated in 384 well plates across an 8-point concentration range in duplicate. A detailed explanation of the multi-parametric analysis of high-content imaging data in this study is provided below under “Quantification and Statistical Analysis.”

#### Defining alternative naive maintenance media

To define the activity of selected hit compounds during extended naive culture, naive hESCs derived from the primed state and maintained in 5i/L/A were dissociated with Accutase (Thermo Fischer Scientific, A1110501) and 1.5 × 10^5^ single cells were seeded in 5i/L/A media on MEF-coated plates. After 24 hours, 5i/L/A media were switched to experimental maintenance conditions to validate hits from the screen. After 4-6 days the cells were split in a 1:2 or 1:3 ratio and further maintained for 4-6 days before analysis by FACS, qRT-PCR, AP staining, and/or RNA-seq. 5i/L/A media in which the MEK inhibitor PD0325901 was replaced with 5 μM AZ628 are represented as alternative 5i/L/A (a5i/L/A). FXGY, AXGY, AXGYU, PXGY, and GXGY media were prepared in serum-free N2B27 media supplemented with 1 μM PD166866, 5 μM AZ628, 5 μM AZ628 and 1 μM UNC0638, 1 μM PD0325901, and 5 μM GDC-0994, respectively, together with 2 μM XAV939, 2 μM Gö6983, and 10 μM Y-27632. During these assays cells were fed with fresh media every second day and cultured in 5% O_2_, 5% CO_2_ at 37°C. Detailed information on conditions used for defining alternative naive maintenance media can be found in Table S3.

#### Primed-to-naive resetting

For primed-to-naive resetting experiments, primed hESCs were dissociated into single cells using TrypLE Express (GIBCO, 12604), washed in fibroblast medium [DMEM (Millipore Sigma, #SLM-021-B) supplemented with 10% FBS (HyClone, SH30396.03, 1X GlutaMAX (GIBCO, 35050), and 1% penicillin-streptomycin (GIBCO, 15140)] and 2 × 10^5^ single primed hESCs were seeded on mitomycin C-inactivated mouse embryonic fibroblast (MEF) feeder cells coated 6 well plates in 3-4 mL mTeSR1 supplemented with 10 μM Y-27632. Two days later, medium was switched to either 5i/L/A or experimental induction media and maintained for 10-12 days. Cells at this stage were considered at passage zero. Cells were then split in a 1:2 or 1:3 ratio every 4-6 days as single cells using Accutase (Thermo Fischer Scientific, A1110501). PXGGY media were prepared in serum-free N2B27 media supplemented with 1 μM PD0325901, 2 μM XAV939, 2 μM Gö6938, 2.5 μM GDC-0994, and 10 μM Y-27632. PXGGY/A media were prepared in PXGGY supplemented with 10 ng/ml Activin A. During these assays cells were fed with fresh media every second day and cultured in 5% O_2_, 5% CO_2_ at 37°C. Detailed information on the conditions used for defining alternative naive induction media can be found in Table S3.

#### Flow cytometry

Primed hESCs were single-cell dissociated using TrypLE Express, while Accutase was used for naive hESCs. Cells were resuspended in fibroblast medium and centrifuged. Cell pellets were washed in 5 mL ice-cold PBS. The cells were then resuspended in 100 mL fresh ice-cold FACS buffer (PBS supplemented with 5% FBS), and incubated with antibodies for 30 minutes on ice in the dark. The following antibodies were used: anti-SUSD2-PE (1:100), anti-CD75-eFluor 660 (1:100), anti-CD90-PE (1:100), anti-ITGA6-FITC (1:100) and EGFR-APC (1:25). Post-incubation the cells were washed once with 1 mL ice-cold PBS, resuspended in fresh 500 μl FACS buffer, and passed through a 0.35 μm cell strainer into round bottom FACS tubes (Corning, #352235). Flow cytometry was performed using a BD LSRFortessa X-20 and the data were analyzed using the FlowJo software.

#### Quantitative reverse transcriptase PCR (qRT-PCR)

Total RNA was isolated using the E.Z.N.A. total RNA kit I and cDNA synthesis was performed from total RNA using the high-capacity cDNA reverse transcription kit (Applied Biosystems, 4368814). qRT-PCR was performed using PowerUp SYBR Green Master Mix (Applied Biosystems, A25743) on the StepOnePlus Real-Time PCR System (Applied Biosystems). Gene expression was normalized to *RPLP0* and analyzed using the ΔCt method. Error bars represent the standard deviation (SD) of the mean of technical replicates.

#### Alkaline phosphatase (AP) staining

AP staining was performed following the manufacturer’s instructions using the Leukocyte Alkaline Phosphatase Kit (Sigma, 86R, 1KT). For AP staining cells were seeded at equal density in in 6-well plates and passaged in the same ratio for all conditions. Post-staining cells were allowed to dry overnight in the dark and scanned using HP Color Laser Jet Managed printer/scanner (MFP E67650).

#### Imaging

The imaging of H9 and WIBR3 hESCs was performed in live conditions within culture media. For WIBR3 *MECP2-GFP/tdTomato* hESCs the medium was aspirated, cell were washed twice with PBS, and imaged in 1 mL of PBS. All images were captured at 10X magnification on a Leica DMi8 microscope.

#### Karyotyping

For G-banded karyotyping naive hESCs were cultured in 6-well plates in 5i/L/A or alternative maintenance media (FXGY, AXGY, PXGY and GXGY) or primed-to-naive resetting medium (PXGGY) for the indicated passage numbers. Cells were then seeded in T25 flasks and karyotyped by the Cytogenetics and Molecular Pathology facility of Washington University in St. Louis using standard methods.

#### Immunoblotting

For western blot analysis of protein expression in primed hESCs, a semi-confluent well was treated with 4 mL of mTeSR1+ROCKi, 5i/L/A, or alternative naive maintenance and induction media for 20 hours. Post-treatment, the cells were washed with 1 mL of cold PBS twice and solubilized in 300 μL of RIPA buffer (Cell Signaling, #9806) with phosphatase inhibitor (ThermoFischer Scientific, #A32957) on ice for ~20 min. For naive hESCs, a single cell dissociation was prepared using Accutase and single cells were transferred to Gelatin-coated plates for 45 minutes at 37°C. This is a feeder-depletion step, which allows the MEFs to attach to the plate surface, while hESCs remain in the media. The media were then collected in 15 mL tubes and centrifuged. Cell pellets were washed twice with 2 mL cold PBS and solubilized in 300 μL RIPA buffer on ice for least 20 min. The lysates from both primed and naive cells were collected after centrifugation. Protein concentration was measured by the Bradford assay (Biorad, #5000006). 20 μg of protein samples were loaded on an SDS-PAGE gel and transferred to a nitrocellulose membrane for immunoblotting. Afterward, the membrane was blocked with 5% nonfat milk (Bio-Rad, #170-6404) at room temperature for 1 hour in TBST (20 mM tris-HCl, pH 7.6, 137 mM NaCl, and 0.1% Tween 20), and incubated with a primary antibody [β-actin (1:2000), ERK1/2 (1:3000), p-ERK1/2 (1:1500), MEK1/2 (1:2500) and p-MEK1/2 (1:1500)] overnight at 4°C, followed by a secondary antibody (1:2000) conjugated with horseradish peroxidase for 45 min at RT. Protein bands on the membrane were detected by the ECL detection system (Biorad #1705060). Immunoblots were imaged and analyzed using the Invitrogen iBright Imaging CL1000 System.

#### RNA sequencing

Total RNA was isolated from 2 million naive or primed cells using the E.Z.N.A. total RNA kit I. Library construction was performed using the SMARTer Directional cDNA Library Construction Kit (Clontech, 634933). Libraries were sequenced on an Illumina Hi-Seq3000 1X50 or NovaSeq S4 2x150 platform at the Genome Technology Access Center at Washington University School of Medicine in St. Louis

#### Whole genome bisulfite sequencing

Genomic DNA was extracted from 2 million naive or primed cells using the DNeasy Blood and Tissue Kit (QIAGEN, Valencia, CA). Whole Genome Bisulfite conversions were performed with 200 ng of gDNA using the EZ DNA Methylation-Gold, 50rxn kit (Fisher, #50444294). WGBS Libraries were created using the Accel-NGS Methyl-Seq DNA Library Kit - 24 rxns (Swift Biosciences, #30024) and Accel-NGS Methyl-Seq Dual Indexing Kit - 96rxns (Swift Biosciences, #38096). The libraries were pooled and sequenced on 0.240 of a NovaSeq S4 flow cell (300 XP; targeting 30x WGBS coverage/120Gb per sample) at the Genome Technology Access Center at Washington University School of Medicine in St. Louis.

#### Re-priming of naive hESCs

For re-priming, a 70%–80% confluent culture of naive hESCs that were maintained in 5i/L/A or alternative conditions were harvested as single cells using Accutase and seeded in a 1:2 ratio on a Matrigel-coated plate in mTeSR1 media supplemented with ROCK inhibitor Y-27632 and cultured in 5% CO_2_ and 20% O_2_ at 37°C. Y-27632 was withdrawn on the second day. Colonies with a flat morphology resembling primed hESCs appeared within 6 days. These colonies were dissociated with TrypLE as single cells and analyzed by FACS for the primed-specific cell surface marker CD90.

#### Capacitation of naive hESCs

Capacitation of naive hESCs was performed as previously described (Rostovskaya et al., 2019). Approximately 0.5 × 10^6^ TrypLE-dissociated naive hESCs were seeded in 5i/L/A or alternative naive conditions on one well of a Geltrex (Thermo Fisher, A1413201) coated 6-well plate. After 48 hours, naive media were switched to capacitation media (N2B27 supplemented with 2 μM XAV939). The cells were fed fresh media every 1-2 days and passaged at 70%–80% confluency (about 4-5 days) using TrypLE (GIBCO, 12604054). 10 μM of Y-27632 was added for 24 hr following passaging. Cells were analyzed for the expression of naive and primed-specific genes by qRT-PCR after 10 days. Capacitation was performed in 5% CO_2_ and 5% O_2_ at 37°C.

#### Derivation of hTSCs from naive hESCs

hTSCs were derived from naive hESCs as previously described (Dong et al., 2020). Briefly, naive hESCs maintained in 5i/L/A or alternative naive media were dissociated into single cells using TrypLE. 0.5-1.0 × 10^6^ cells were seeded in a 6-well plate pre-coated with 5 μg/mL Collagen IV and switched to 2 mL hTSC medium (Okae et al., 2018) [DMEM/F12 supplemented with 0.1 mM 2-mercaptoethanol, 0.2% FBS, 0.5% Penicillin-Streptomycin, 0.3% BSA, 1% ITS-X (GIBCO, 51500), 1.5 μg/ml L-ascorbic acid (Wako, 013–12061), 50 ng/ml EGF (Rockland, 009–001 C26), 2 μM CHIR99021 (Stemgent, 04–0004), 0.5 μM A83-01 (BioVision, 1725), 1 μM SB431542 (BioVision, 1674), 0.8 mM VPA (Tocris, 2815), and 5 μM Y-27632]. Cells were cultured in 5% CO_2_ and 20% O_2_ at 37°C, media were changed every 2 days, and passaged upon reaching 80%–100% confluency at a ratio of 1:2 to 1:4 using TrypLE. Cells were analyzed by FACS for the hTSC-specific cell surface markers ITGA6 and EGFR after 5-7 passages.

### QUANTIFICATION AND STATISTICAL ANALYSIS

#### Multi-parametric analysis of high-content imaging data

Images were analyzed using a Yokogawa Cell Voyager 7000 High-Content Imaging System. A total of 112 features were calculated from the data and these included statistics (e.g., mean, median, total, sum, standard deviations, covariance) and maximum and minimum values of fluorescent object shape parameters (e.g., area, circumference, diameter, circularity, anisometry, and compactness) as well as fluorescent intensity values (e.g., mean, median, total, maximum, and minimum). To determine which set of parameters provided the most robust Z′-factors (a measure of statistical effect size, see below) we applied a Multi-Parametric Data Analysis (MPDA) algorithm to analyze these images, selecting features showing robust Z′-factors taken from the calculated shape and fluorescent intensity parameters. We then computed a Mahalanobis distance score (MHD) between each compound and the active (+DOX) and negative (−DOX) control wells based on features that included area, compactness and fluorescence intensity (typically a combination of shape parameters such as area and mean intensity provided acceptable Z′-factors) (Zhang et al., 1999). This provides a multivariate generalization to determine the similarity between controls (+/−DOX) and compound-treated wells and distinguished objects that were likely due to cell death and phenotypes that were disparate from the desired phenotype observed in the positive control wells (Figure S1A).

The Z’-factor is a measure of statistical effect size used to assess the robustness of a high-throughput screen based on the means and standard deviations of both the positive and negative controls. The Z′-factor was calculated as:

$$Z^{\prime }=\frac{3\sigma_{+}+3\sigma_{-}}{|\mu_{+}-\mu_{-}|}$$

Where σ_+_ = standard deviation of the MHD score for +DOX-treated wells

σ_−_ = standard deviation of the MHD score of −DOX-treated wells

μ_+_ = average MHD score for +DOX-treated wells

μ_−_ = average MHD score for −DOX-treated wells

Hits were then called based on those sample wells showing an MHD value similar to the +DOX control wells by employing a threshold of > 1 SD from the MHD value of the −DOX control wells in duplicate runs (Figure S1A). Additionally to further refine the hit list, all the raw images and calculated data were visualized using TIBICO Spotfire where the images from sample wells designated as hits could be easily compared to positive and negative control wells.

#### RNA-seq data analysis

RNA-seq reads were aligned to the human genome hg38 with HISAT2 version 2.2.0 (Kim et al., 2019). Gene counts were derived from the number of uniquely aligned unambiguous reads by Subread:featureCount (Liao et al., 2014), version 1.4.6, with hg38 annotation gencodeV31 (Harrow et al., 2012). All gene-level transcript counts were then imported into the R/Bioconductor package DESeq2 (Love et al., 2014). Transcripts with CPM > 1.0 were converted into a DESeq2 dataset and then regularized log transformed using the rlog function from the DESeq2 package. Adjusted p values for DGE were determined by DESeq2 using the R stats function p.adjust using the Benjamini and Hochberg correction to determine the false discovery rate with a 1.5-fold expression change and FDR < 0.05 required to consider a gene differentially expressed. Dimension reduction using Uniform Manifold Approximation (UMAP) and comparison with single-cell RNA-seq data was performed using the R/Bioconductor package Seurat and plotted using the R package scatterplot3D.

#### Whole genome bisulfite sequencing data analysis

WGBSdata were trimmed of adapters using Cutadapt version 1.2.1 (Martin, 2011), and aligned to the human genome hg38 with Bismark version 0.22.3 (Krueger and Andrews, 2011). The aligned reads were further processed with MethPipe (Song et al., 2013), and % DNA methylation levels of CpGs on imprinted regions were compiled with the roimethstats function from MethPipe. The genome-wide average % DNA methylation of CpGs was compiled on 1 kB sliding windows (offset 500 bp).

## Supplementary Material

1

2

3

4

5

## Figures and Tables

**Table:** KEY RESOURCES TABLE

REAGENT or RESOURCE	SOURCE	IDENTIFIER
Antibodies		
anti-SUSD2-PE	BioLegend	Cat# 327406; RRID: AB_940654
anti-CD75-eFluor 660	Thermo Fischer	Cat# 50-0759-42; RRID: AB_2574175
anti-CD90-PE	BioLegend	Cat# 328110; RRID: AB_893433
anti-ITGA6-FITC	Miltenyi Biotec	Cat#130-097-245; RRID: AB_2658552
anti-EGFR-APC	BioLegend	Cat# 322905; RRID: AB_11148943
anti-ERK1/2	Cell signaling	Cat# 4696; RRID: AB_390780
anti-p-ERK1/2	Cell signaling	Cat# 4370, RRID:AB_2315112
anti-MEK1/2	Cell signaling	Cat# 9122, RRID:AB_823567
anti-p-MEK1/2	Cell signaling	Cat# 9121; RRID:AB_331648
anti-β-actin	Cell signaling	Cat# 4970; RRID:AB_2223172
Chemicals, peptides, and recombinant proteins		
1-Naphthyl PP1	Tocris	3063
Semapimod	Chemspace	EN300-261068
Laccaic acid/ Natural red 25	Sigma	50506
PD166866	Selleckchem	S8493
UNC0638	Tocris	434310
AZ628	Selleckchem	S2746
Brequinar	Sigma	SML0113
EHT 1864	Tocris	387210
LDC000067	Selleckchem	S7461
LY364947	Selleckchem	S2805
OF-1	Selleckchem	S7681
Vorapaxar	Selleckchem	S8067
GDC-0994	Selleckchem	S7554
Y-27632	Stemgent	04–0012
PD0325901	Stemgent	04–0006
IM-12	Enzo	BML-WN102
SB590885	Tocris	2650
WH-4-023	A Chemtek	H620061
XAV939	Sigma	X3004
Gö6983	Tocris	2285
ICI 118,551 hydrochloride	Sigma	I127
BMX-IN-1	Tocris	5123
J113863	Tocris	2595
NSC 109555 ditosylate	Tocris	3034
Erlotinib	Selleckchem	S7786
Sapitinib	Selleckchem	S2192
MPEP Hydrochloride	Tocris	1212
GSK2033	Med Chem Express	HY-108688
GS-493	Sigma	538099
Activin A	PeproTech	120-14
LIF	PeproTech	300-05
Critical commercial assays		
Leukocyte Alkaline Phosphatase Kit	Sigma	86R-1KY
E.Z.N.A. total RNA kit	Omega	D6834
DNeasy Blood and Tissue Kit	QIAGEN	69504
Deposited data		
Raw and Processed data	This paper	GEO: GSE153215
A developmental landscape of 3D-cultured human pre-gastrulation embryos	Xiang et al., 2020	GEO: GSE136447
A developmental coordinate of pluripotency among mice, monkeys and humans	Nakamura et. al, 2016	GEO: GSE74767
Capacitation of human naive pluripotent stem cells for multi-lineage differentiation	Rostovskaya et al., 2019	GEO: GSE123055
Experimental models: Cell lines		
H9 (hESC)	WashU GEiC	N/A
WIBR3 (hESC)	Whitehead Institute	N/A
WIBR3 *OCT4-ΔPE-GFP* (hESC)	Whitehead Institute	N/A
WIBR3 *MECP2-GFP/tdTomato* (hESC)	Whitehead Institute	N/A
Oligonucleotides		
RPLP0-F: GCTTCCTGGAGGGTGTCC	This paper	N/A
RPLP0-R: GGACTCGTTTGTACCCGTTG	This paper	N/A
KLF17-F: CTGCCTGAGCGTGGTATGAG	This paper	N/A
KLF17-R: TCATCCGGGAAGGAGTGAGA	This paper	N/A
DNMT3L-F: TTCTGGATGTTCGTGGACAA	This paper	N/A
DNMT3L-R: ACATCTGGGATGGTGACTGG	This paper	N/A
ZIC2-F: CCCTTCAAGGCCAAATACAA	This paper	N/A
ZIC2-R: TGCATGTGCTTCTTCCTGTC	This paper	N/A
SFRP2-F: ACGGCATCGAATACCAGAACA	This paper	N/A
SFRP2-R: CTCGTCTAGGTCATCGAGGCA	This paper	N/A
VIM-F: TGTCCAAATCGATGTGGATGTTTC	This paper	N/A
VIM-R: TTGTACCATTCTTCTGCCTCCTG	This paper	N/A
eGFP-F: CGACCACTACCAGCAGAACA	This paper	N/A
eGFP-R: GAACTCCAGCAGGACCATGT	This paper	N/A
Software and algorithms		
FlowJo_v10.6.2	FlowJo	RRID:SCR_008520; https://www.flowjo.com/
Prism 8	GraphPad	RRID:SCR_002798; https://www.graphpad.com
R 4.0.0	R project	N/A; https://www.r-project.org/
Bowtie2 2.4.2	Github	RRID:SCR_016368; https://github.com/BenLangmead/bowtie2/releases
DeSeq2 1.30.1	Bioconductor	RRID:SCR_017673; https://bioconductor.org/packages/release/bioc/html/DESeq2.html
Bismark 0.23.0	Babraham Institute	RRID:SCR_005604; https://www.bioinformatics.babraham.ac.uk/projects/bismark/
MethPipe 4.1.1	Andrew Smith lab	RRID:SCR_005168; http://smithlabresearch.org/software/methpipe/

## Highlights

- Chemical screening for compounds that maintain naive hESCs in minimal conditions
- MEK inhibitors can be replaced by FGFR, RAF, or ERK inhibitors in naive hESCs
- Alternative naive hESCs exhibit elevated p-ERK levels and HERVH transcription
- Dual MEK/ERK inhibition promotes efficient primed-to-naive resetting with activin A

## References

[R1] An C, Feng G, Zhang J, Cao S, Wang Y, Wang N, Lu F, Zhou Q, and Wang H (2020). Overcoming Autocrine FGF Signaling-Induced Heterogeneity in Naive Human ESCs Enables Modeling of Random X Chromosome Inactivation. Cell Stem Cell 27, 482–497. 32673569 10.1016/j.stem.2020.06.002

[R2] Basken J, Stuart SA, Kavran AJ, Lee T, Ebmeier CC, Old WM, and Ahn NG (2018). Specificity of Phosphorylation Responses to Mitogen Activated Protein (MAP) Kinase Pathway Inhibitors in Melanoma Cells. Mol. Cell. Proteomics 17, 550–564. 29255136 10.1074/mcp.RA117.000335 PMC5880111

[R3] Blakeley P, Fogarty NME, del Valle I, Wamaitha SE, Hu TX, Elder K, Snell P, Christie L, Robson P, and Niakan KK (2015). Defining the three cell lineages of the human blastocyst by single-cell RNA-seq. Development 142, 3151–3165. 26293300 10.1242/dev.123547 PMC4582176

[R4] Boroviak T, Loos R, Lombard P, Okahara J, Behr R, Sasaki E, Nichols J, Smith A, and Bertone P (2015). Lineage-Specific Profiling Delineates the Emergence and Progression of Naive Pluripotency in Mammalian Embryogenesis. Dev. Cell 35, 366–382. 26555056 10.1016/j.devcel.2015.10.011 PMC4643313

[R5] Bredenkamp N, Stirparo GG, Nichols J, Smith A, and Guo G (2019a). The Cell-Surface Marker Sushi Containing Domain 2 Facilitates Establishment of Human Naive Pluripotent Stem Cells. Stem Cell Rep. 12, 1212–1222. 10.1016/j.stemcr.2019.03.014 PMC6565611 31031191

[R6] Bredenkamp N, Yang J, Clarke J, Stirparo GG, von Meyenn F, Dietmann S, Baker D, Drummond R, Ren Y, Li D, (2019b). Wnt Inhibition Facilitates RNA-Mediated Reprogramming of Human Somatic Cells to Naive Pluripotency. Stem Cell Reports 13, 1083–1098. 31708477 10.1016/j.stemcr.2019.10.009 PMC6915845

[R7] Brons IG, Smithers LE, Trotter MW, Rugg-Gunn P, Sun B, Chuva de Sousa Lopes SM, Howlett SK, Clarkson A, Ahrlund-Richter L, Pedersen RA, and Vallier L (2007). Derivation of pluripotent epiblast stem cells from mammalian embryos. Nature 448, 191–195. 17597762 10.1038/nature05950

[R8] Canham SM, Wang Y, Cornett A, Auld DS, Baeschlin DK, Patoor M, Skaanderup PR, Honda A, Llamas L, Wendel G, (2020). Systematic Chemogenetic Library Assembly. Cell Chem. Biol 27, 1124–1129. 32707038 10.1016/j.chembiol.2020.07.004

[R9] Castel G, Meistermann D, Bretin B, Firmin J, Blin J, Loubersac S, Bruneau A, Chevolleau S, Kilens S, Chariau C, (2020). Induction of Human Trophoblast Stem Cells from Somatic Cells and Pluripotent Stem Cells. Cell Rep. 33, 108419. 33238118 10.1016/j.celrep.2020.108419

[R10] Chan YS, Göke J, Ng JH, Lu X, Gonzales KA, Tan CP, Tng WQ, Hong ZZ, Lim YS, and Ng HH (2013). Induction of a human pluripotent state with distinct regulatory circuitry that resembles preimplantation epiblast. Cell Stem Cell 13, 663–675. 24315441 10.1016/j.stem.2013.11.015

[R11] Choi J, Huebner AJ, Clement K, Walsh RM, Savol A, Lin K, Gu H, Di Stefano B, Brumbaugh J, Kim SY, (2017). Prolonged Mek1/2 suppression impairs the developmental potential of embryonic stem cells. Nature 548, 219–223. 28746311 10.1038/nature23274 PMC5905676

[R12] Cinkornpumin JK, Kwon SY, Guo Y, Hossain I, Sirois J, Russett CS, Tseng HW, Okae H, Arima T, Duchaine TF, (2020). Naive Human Embryonic Stem Cells Can Give Rise to Cells with a Trophoblast-like Transcriptome and Methylome. Stem Cell Reports 15, 198–213. 32619492 10.1016/j.stemcr.2020.06.003 PMC7363941

[R13] Collier AJ, Panula SP, Schell JP, Chovanec P, Plaza Reyes A, Petropoulos S, Corcoran AE, Walker R, Douagi I, Lanner F, and Rugg-Gunn PJ (2017). Comprehensive Cell Surface Protein Profiling Identifies Specific Markers of Human Naive and Primed Pluripotent States. Cell Stem Cell 20, 874–890. 28343983 10.1016/j.stem.2017.02.014 PMC5459756

[R14] Cornacchia D, Zhang C, Zimmer B, Chung SY, Fan Y, Soliman MA, Tchieu J, Chambers SM, Shah H, Paull D, (2019). Lipid Deprivation Induces a Stable, Naive-to-Primed Intermediate State of Pluripotency in Human PSCs. Cell Stem Cell 25, 120–136. 31155483 10.1016/j.stem.2019.05.001 PMC7549840

[R15] Di Stefano B, Ueda M, Sabri S, Brumbaugh J, Huebner AJ, Sahakyan A, Clement K, Clowers KJ, Erickson AR, Shioda K, (2018). Reduced MEK inhibition preserves genomic stability in naive human embryonic stem cells. Nat. Methods 15, 732–740. 30127506 10.1038/s41592-018-0104-1 PMC6127858

[R16] Dong C, Beltcheva M, Gontarz P, Zhang B, Popli P, Fischer LA, Khan SA, Park KM, Yoon EJ, Xing X, (2020). Derivation of trophoblast stem cells from naïve human pluripotent stem cells. eLife 9, e52504. 32048992 10.7554/eLife.52504 PMC7062471

[R17] Gafni O, Weinberger L, Mansour AA, Manor YS, Chomsky E, Ben-Yosef D, Kalma Y, Viukov S, Maza I, Zviran A, (2013). Derivation of novel human ground state naive pluripotent stem cells. Nature 504, 282–286. 24172903 10.1038/nature12745

[R18] Giulitti S, Pellegrini M, Zorzan I, Martini P, Gagliano O, Mutarelli M, Ziller MJ, Cacchiarelli D, Romualdi C, Elvassore N, and Martello G (2019). Direct generation of human naive induced pluripotent stem cells from somatic cells in microfluidics. Nat. Cell Biol 21, 275–286. 30598530 10.1038/s41556-018-0254-5

[R19] Guo H, Zhu P, Yan L, Li R, Hu B, Lian Y, Yan J, Ren X, Lin S, Li J, (2014). The DNA methylation landscape of human early embryos. Nature 511, 606–610. 25079557 10.1038/nature13544

[R20] Guo G, von Meyenn F, Santos F, Chen Y, Reik W, Bertone P, Smith A, and Nichols J (2016). Naive Pluripotent Stem Cells Derived Directly from Isolated Cells of the Human Inner Cell Mass. Stem Cell Reports 6, 437–446. 26947977 10.1016/j.stemcr.2016.02.005 PMC4834040

[R21] Guo G, von Meyenn F, Rostovskaya M, Clarke J, Dietmann S, Baker D, Sahakyan A, Myers S, Bertone P, Reik W, (2017). Epigenetic resetting of human pluripotency. Development 144, 2748–2763. 28765214 10.1242/dev.146811 PMC5560041

[R22] Guo G, Stirparo GG, Strawbridge SE, Spindlow D, Yang J, Clarke J, Dattani A, Yanagida A, Li MA, Myers S, (2021). Human naive epiblast cells possess unrestricted lineage potential. Cell Stem Cell 28, 1–17. 33831366 10.1016/j.stem.2021.02.025 PMC8189439

[R23] Hackett JA, Dietmann S, Murakami K, Down TA, Leitch HG, and Surani MA (2013). Synergistic mechanisms of DNA demethylation during transition to ground-state pluripotency. Stem Cell Reports 1, 518–531. 24371807 10.1016/j.stemcr.2013.11.010 PMC3871394

[R24] Hanna J, Cheng AW, Saha K, Kim J, Lengner CJ, Soldner F, Cassady JP, Muffat J, Carey BW, and Jaenisch R (2010). Human embryonic stem cells with biological and epigenetic characteristics similar to those of mouse ESCs. Proc. Natl. Acad. Sci. USA 107, 9222–9227. 20442331 10.1073/pnas.1004584107 PMC2889088

[R25] Harrow J, Frankish A, Gonzalez JM, Tapanari E, Diekhans M, Kokocinski F, Aken BL, Barrell D, Zadissa A, Searle S, (2012). GENCODE: the reference human genome annotation for The ENCODE Project. Genome Res. 22, 1760–1774. 22955987 10.1101/gr.135350.111 PMC3431492

[R26] Huang K, Maruyama T, and Fan G (2014). The naive state of human pluripotent stem cells: a synthesis of stem cell and preimplantation embryo transcriptome analyses. Cell Stem Cell 15, 410–415. 25280217 10.1016/j.stem.2014.09.014 PMC5507179

[R27] Io S, Kabata M, Iemura Y, Semi K, Morone N, Minagawa A, Wang B, Okamoto I, Nakamura T, Kojima Y, (2021). Capturing human trophoblast development with naive pluripotent stem cells in vitro. Cell Stem Cell, Published online 4 5, 2021. 10.1016/j.stem.2021.03.013. 33831365

[R28] Kilens S, Meistermann D, Moreno D, Chariau C, Gaignerie A, Reignier A, Lelièvre Y, Casanova M, Vallot C, Nedellec S, ; Milieu Intérieur Consortium (2018). Parallel derivation of isogenic human primed and naive induced pluripotent stem cells. Nat. Commun 9, 360. 29367672 10.1038/s41467-017-02107-w PMC5783949

[R29] Kim D, Paggi JM, Park C, Bennett C, and Salzberg SL (2019). Graph-based genome alignment and genotyping with HISAT2 and HISAT-genotype. Nat. Biotechnol 37, 907–915. 31375807 10.1038/s41587-019-0201-4 PMC7605509

[R30] Kojima Y, Kaufman-Francis K, Studdert JB, Steiner KA, Power MD, Loebel DA, Jones V, Hor A, de Alencastro G, Logan GJ, (2014). The transcriptional and functional properties of mouse epiblast stem cells resemble the anterior primitive streak. Cell Stem Cell 14, 107–120. 24139757 10.1016/j.stem.2013.09.014

[R31] Krueger F, and Andrews SR (2011). Bismark: a flexible aligner and methylation caller for Bisulfite-Seq applications. Bioinformatics 27, 1571–1572. 21493656 10.1093/bioinformatics/btr167 PMC3102221

[R32] Lake D, Corrêa SA, and Müller J (2016). Negative feedback regulation of the ERK1/2 MAPK pathway. Cell. Mol. Life Sci 73, 4397–4413. 27342992 10.1007/s00018-016-2297-8 PMC5075022

[R33] Lau KX, Mason EA, Kie J, De Souza DP, Kloehn J, Tull D, McConville MJ, Keniry A, Beck T, Blewitt ME, (2020). Unique properties of a subset of human pluripotent stem cells with high capacity for self-renewal. Nat. Commun 11, 2420. 32415101 10.1038/s41467-020-16214-8 PMC7229198

[R34] Lee HJ, Hore TA, and Reik W (2014). Reprogramming the methylome: erasing memory and creating diversity. Cell Stem Cell 14, 710–719. 24905162 10.1016/j.stem.2014.05.008 PMC4051243

[R35] Leitch HG, McEwen KR, Turp A, Encheva V, Carroll T, Grabole N, Mansfield W, Nashun B, Knezovich JG, Smith A, (2013). Naive pluripotency is associated with global DNA hypomethylation. Nat. Struct. Mol. Biol 20, 311–316. 23416945 10.1038/nsmb.2510 PMC3591483

[R36] Liao Y, Smyth GK, and Shi W (2014). featureCounts: an efficient general purpose program for assigning sequence reads to genomic features. Bioinformatics 30, 923–930. 24227677 10.1093/bioinformatics/btt656

[R37] Linneberg-Agerholm M, Wong YF, Romero Herrera JA, Monteiro RS, Anderson KGV, and Brickman JM (2019). Naïve human pluripotent stem cells respond to Wnt, Nodal and LIF signalling to produce expandable naïve extra-embryonic endoderm. Development 146, 146. 10.1242/dev.180620 31740534

[R38] Liu X, Nefzger CM, Rossello FJ, Chen J, Knaupp AS, Firas J, Ford E, Pflueger J, Paynter JM, Chy HS, (2017). Comprehensive characterization of distinct states of human naive pluripotency generated by reprogramming. Nat. Methods 14, 1055–1062. 28945704 10.1038/nmeth.4436

[R39] Love MI, Huber W, and Anders S (2014). Moderated estimation of fold change and dispersion for RNA-seq data with DESeq2. Genome Biol. 15, 550. 25516281 10.1186/s13059-014-0550-8 PMC4302049

[R40] Martin M (2011). Cutadapt Removes Adapter Sequences from High-Throughput Sequencing Reads (Embnet).

[R41] Nakamura T, Okamoto I, Sasaki K, Yabuta Y, Iwatani C, Tsuchiya H, Seita Y, Nakamura S, Yamamoto T, and Saitou M (2016). A developmental coordinate of pluripotency among mice, monkeys and humans. Nature 537, 57–62. 27556940 10.1038/nature19096

[R42] Nakanishi M, Mitchell RR, Benoit YD, Orlando L, Reid JC, Shimada K, Davidson KC, Shapovalova Z, Collins TJ, Nagy A, and Bhatia M (2019). Human Pluripotency Is Initiated and Preserved by a Unique Subset of Founder Cells. Cell 177, 910–924. 30982595 10.1016/j.cell.2019.03.013

[R43] Nichols J, and Smith A (2009). Naive and primed pluripotent states. Cell Stem Cell 4, 487–492. 19497275 10.1016/j.stem.2009.05.015

[R44] Okae H, Toh H, Sato T, Hiura H, Takahashi S, Shirane K, Kabayama Y, Suyama M, Sasaki H, and Arima T (2018). Derivation of Human Trophoblast Stem Cells. Cell Stem Cell 22, 50–63. 29249463 10.1016/j.stem.2017.11.004

[R45] Pastor WA, Chen D, Liu W, Kim R, Sahakyan A, Lukianchikov A, Plath K, Jacobsen SE, and Clark AT (2016). Naive Human Pluripotent Cells Feature a Methylation Landscape Devoid of Blastocyst or Germline Memory. Cell Stem Cell 18, 323–329. 26853856 10.1016/j.stem.2016.01.019 PMC4779431

[R46] Pegram LM, Liddle JC, Xiao Y, Hoh M, Rudolph J, Iverson DB, Vigers GP, Smith D, Zhang H, Wang W, (2019). Activation loop dynamics are controlled by conformation-selective inhibitors of ERK2. Proc. Natl. Acad. Sci. USA 116, 15463–15468. 31311868 10.1073/pnas.1906824116 PMC6681744

[R47] Pontis J, Planet E, Offner S, Turelli P, Duc J, Coudray A, Theunissen TW, Jaenisch R, and Trono D (2019). Hominoid-Specific Transposable Elements and KZFPs Facilitate Human Embryonic Genome Activation and Control Transcription in Naive Human ESCs. Cell Stem Cell 24, 724–735. 31006620 10.1016/j.stem.2019.03.012 PMC6509360

[R48] Rostovskaya M, Stirparo GG, and Smith A (2019). Capacitation of human naïve pluripotent stem cells for multi-lineage differentiation. Development 146, dev172916. 30944104 10.1242/dev.172916 PMC6467473

[R49] Sahakyan A, Kim R, Chronis C, Sabri S, Bonora G, Theunissen TW, Kuoy E, Langerman J, Clark AT, Jaenisch R, and Plath K (2017). Human Naive Pluripotent Stem Cells Model X Chromosome Dampening and X Inactivation. Cell Stem Cell 20, 87–101. 27989770 10.1016/j.stem.2016.10.006 PMC5218861

[R50] Song Q, Decato B, Hong EE, Zhou M, Fang F, Qu J, Garvin T, Kessler M, Zhou J, and Smith AD (2013). A reference methylome database and analysis pipeline to facilitate integrative and comparative epigenomics. PLoS ONE 8, e81148. 24324667 10.1371/journal.pone.0081148 PMC3855694

[R51] Stirparo GG, Boroviak T, Guo G, Nichols J, Smith A, and Bertone P (2018). Integrated analysis of single-cell embryo data yields a unified transcriptome signature for the human pre-implantation epiblast. Development 145, dev158501. 29361568 10.1242/dev.158501 PMC5818005

[R52] Takashima Y, Guo G, Loos R, Nichols J, Ficz G, Krueger F, Oxley D, Santos F, Clarke J, Mansfield W, (2014). Resetting transcription factor control circuitry toward ground-state pluripotency in human. Cell 158, 1254–1269. 25215486 10.1016/j.cell.2014.08.029 PMC4162745

[R53] Tesar PJ, Chenoweth JG, Brook FA, Davies TJ, Evans EP, Mack DL, Gardner RL, and McKay RD (2007). New cell lines from mouse epiblast share defining features with human embryonic stem cells. Nature 448, 196–199. 17597760 10.1038/nature05972

[R54] Theunissen TW, Powell BE, Wang H, Mitalipova M, Faddah DA, Reddy J, Fan ZP, Maetzel D, Ganz K, Shi L, (2014). Systematic identification of culture conditions for induction and maintenance of naive human pluripotency. Cell Stem Cell 15, 471–487. 25090446 10.1016/j.stem.2014.07.002 PMC4184977

[R55] Theunissen TW, Friedli M, He Y, Planet E, O’Neil RC, Markoulaki S, Pontis J, Wang H, Iouranova A, Imbeault M, (2016). Molecular Criteria for Defining the Naive Human Pluripotent State. Cell Stem Cell 19, 502–515. 27424783 10.1016/j.stem.2016.06.011 PMC5065525

[R56] Vallot C, Patrat C, Collier AJ, Huret C, Casanova M, Liyakat Ali TM, Tosolini M, Frydman N, Heard E, Rugg-Gunn PJ, and Rougeulle C (2017). XACT Noncoding RNA Competes with XIST in the Control of X Chromosome Activity during Human Early Development. Cell Stem Cell 20, 102–111. 27989768 10.1016/j.stem.2016.10.014 PMC5222720

[R57] von Meyenn F, Iurlaro M, Habibi E, Liu NQ, Salehzadeh-Yazdi A, Santos F, Petrini E, Milagre I, Yu M, Xie Z, (2016). Impairment of DNA Methylation Maintenance Is the Main Cause of Global Demethylation in Naive Embryonic Stem Cells. Mol. Cell 62, 848–861. 27237052 10.1016/j.molcel.2016.04.025 PMC4914828

[R58] Wang Y, Zhao C, Hou Z, Yang Y, Bi Y, Wang H, Zhang Y, and Gao S (2018). Unique molecular events during reprogramming of human somatic cells to induced pluripotent stem cells (iPSCs) at naïve state. eLife 7, e29518. 29381138 10.7554/eLife.29518 PMC5807049

[R59] Ware CB, Nelson AM, Mecham B, Hesson J, Zhou W, Jonlin EC, Jimenez-Caliani AJ, Deng X, Cavanaugh C, Cook S, (2014). Derivation of naive human embryonic stem cells. Proc. Natl. Acad. Sci. USA 111, 4484–4489. 24623855 10.1073/pnas.1319738111 PMC3970494

[R60] Watanabe K, Ueno M, Kamiya D, Nishiyama A, Matsumura M, Wataya T, Takahashi JB, Nishikawa S, Nishikawa S, Muguruma K, and Sasai Y (2007). A ROCK inhibitor permits survival of dissociated human embryonic stem cells. Nat. Biotechnol 25, 681–686. 17529971 10.1038/nbt1310

[R61] Wenglowsky S, Moreno D, Laird ER, Gloor SL, Ren L, Risom T, Rudolph J, Sturgis HL, and Voegtli WC (2012). Pyrazolopyridine inhibitors of B-Raf(V600E). Part 4: rational design and kinase selectivity profile of cell potent type II inhibitors. Bioorg. Med. Chem. Lett 22, 6237–6241. 22954737 10.1016/j.bmcl.2012.08.007

[R62] Wu J, Greely HT, Jaenisch R, Nakauchi H, Rossant J, and Belmonte JC (2016). Stem cells and interspecies chimaeras. Nature 540, 51–59. 27905428 10.1038/nature20573

[R63] Xiang L, Yin Y, Zheng Y, Ma Y, Li Y, Zhao Z, Guo J, Ai Z, Niu Y, Duan K, (2020). A developmental landscape of 3D-cultured human pre-gastrulation embryos. Nature 577, 537–542. 31830756 10.1038/s41586-019-1875-y

[R64] Yagi M, Kishigami S, Tanaka A, Semi K, Mizutani E, Wakayama S, Wakayama T, Yamamoto T, and Yamada Y (2017). Derivation of ground-state female ES cells maintaining gamete-derived DNA methylation. Nature 548, 224–227. 28746308 10.1038/nature23286

[R65] Ying QL, Wray J, Nichols J, Batlle-Morera L, Doble B, Woodgett J, Cohen P, and Smith A (2008). The ground state of embryonic stem cell self-renewal. Nature 453, 519–523. 18497825 10.1038/nature06968 PMC5328678

[R66] Yu L, Wei Y, Duan J, Schmitz DA, Sakurai M, Wang L, Wang K, Zhao S, Hon GC, and Wu J (2021). Blastocyst-like structures generated from human pluripotent stem cells. Nature 591, 620–626. 33731924 10.1038/s41586-021-03356-y

[R67] Zhang JH, Chung TD, and Oldenburg KR (1999). A Simple Statistical Parameter for Use in Evaluation and Validation of High Throughput Screening Assays. J. Biomol. Screen 4, 67–73. 10838414 10.1177/108705719900400206

[R68] Zimmerlin L, Park TS, Huo JS, Verma K, Pather SR, Talbot CC Jr., Agarwal J, Steppan D, Zhang YW, Considine M, (2016). Tankyrase inhibition promotes a stable human naïve pluripotent state with improved functionality. Development 143, 4368–4380. 27660325 10.1242/dev.138982 PMC5201042

