# Supplementary material for: Probing the signaling requirements for naive human pluripotency by high-throughput chemical screening
Source: Cell Rep. Author manuscript; Available in PMC 2021 Jul 10. (PMC8272458; doi:10.1016/j.celrep.2021.109233)
Supplement: 1 [file NIHMS1716898-supplement-1.pdf]

Cell Reports, Volume *35*

Supplemental information

Probing the signaling requirements

for naive human pluripotency

by high-throughput chemical screening

Shafqat A. Khan, Kyoung-mi Park, Laura A. Fischer, Chen Dong, Tenzin Lungjangwa, Marta Jimenez, Dominick Casalena, Brian Chew, Sabine Dietmann, Douglas S. Auld, Rudolf Jaenisch, and Thorold W. Theunissen

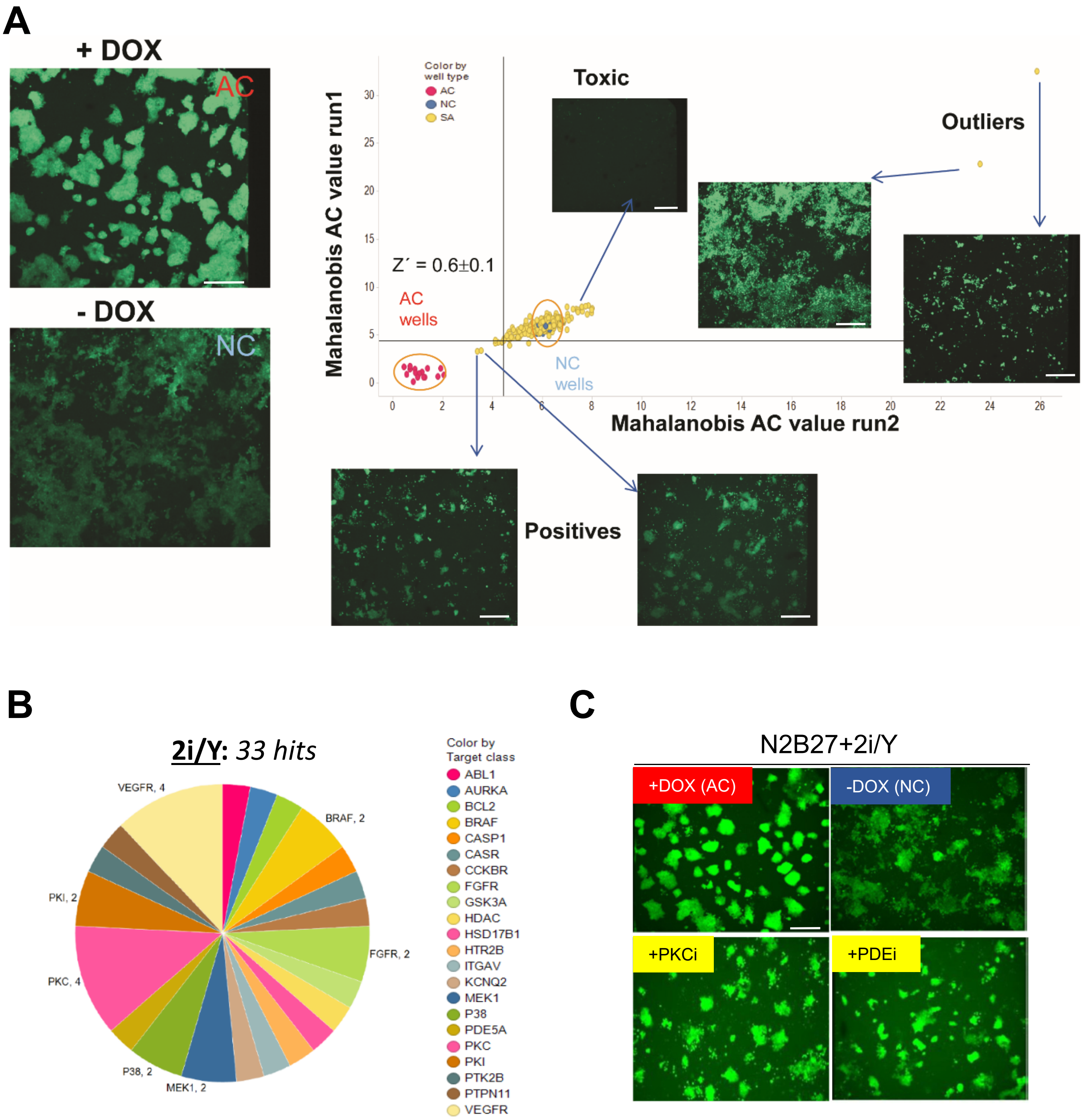
A
+ DOX
AC
- DOX
NC
Color by well type
AC
NC
SA
Toxic
Outliers
Z´ = 0.6±0.1
AC wells
NC wells
Mahalanobis AC value run1
Mahalanobis AC value run2
Positives
B
2i/Y: 33 hits
VEGFR, 4
BRAF, 2
PKI, 2
FGFR, 2
PKC, 4
P38, 2
MEK1, 2
Color by Target class
ABL1
AURKA
BCL2
BRAF
CASP1
CASR
CCKBR
FGFR
GSK3A
HDAC
HSD17B1
HTR2B
ITGAV
KCNQ2
MEK1
P38
PDE5A
PKC
PKI
PTK2B
PTPN11
VEGFR
C
N2B27+2i/Y
+DOX (AC)
-DOX (NC)
+PKCi
+PDEi

Figure S1

Figure S1. High-throughput chemical screening for modulators of naive human pluripotency in the presence of 2i [Related to Figure 1].

A. Illustration of the multi-parametric data analysis (MPDA) for high-throughput screening in this study. Shown is an example dataset from a 5 µM compound screening concentration in presence of MEK, GSK3, and ROCK inhibitors (2i/Y) in two biological replicates. Following the extraction of fluorescent object shape and intensity statistics, the Mahalanobis distance (MHD) scores were calculated relative to the +DOX control wells (active control = AC wells) and hits were called based on those sample wells that showed >1 SD from the –DOX control wells (negative control = NC wells). Use of the MHD scores allowed enumeration of unwanted phenotypes that showed, for example, very bright fluorescence but poor shape parameters such as the outlier wells shown that may include compounds showing toxicity leading to very bright and round but small objects or those where GFP expression is increased without the desired roundness observed in the AC wells. Lines on the graph represent 1 SD of the NC wells. Fluorescent images are included of active control (+DOX), negative control (-DOX), and selected hit compounds in presence of 2i/Y. The scale bar depicts 260 µm.

B. Summary of 33 validated hit compounds from the MoA library screen performed in presence of 2i/Y. Compound target annotations showed enrichments in several target classes, some of which have been previously been implicated in naive human pluripotency. Novel putative targets were also identified. Numbers represent the number of compounds identified that were annotated to the target noted. Hit compounds were validated in two biological replicates. A full list of validated hit compounds in presence of 2i/Y is included in Table S1.

C. Fluorescent images of active control (+DOX), negative control (-DOX), and selected hit compounds in presence of 2i/Y. Hit compounds were validated in two biological replicates. The scale bar depicts 260 µm.

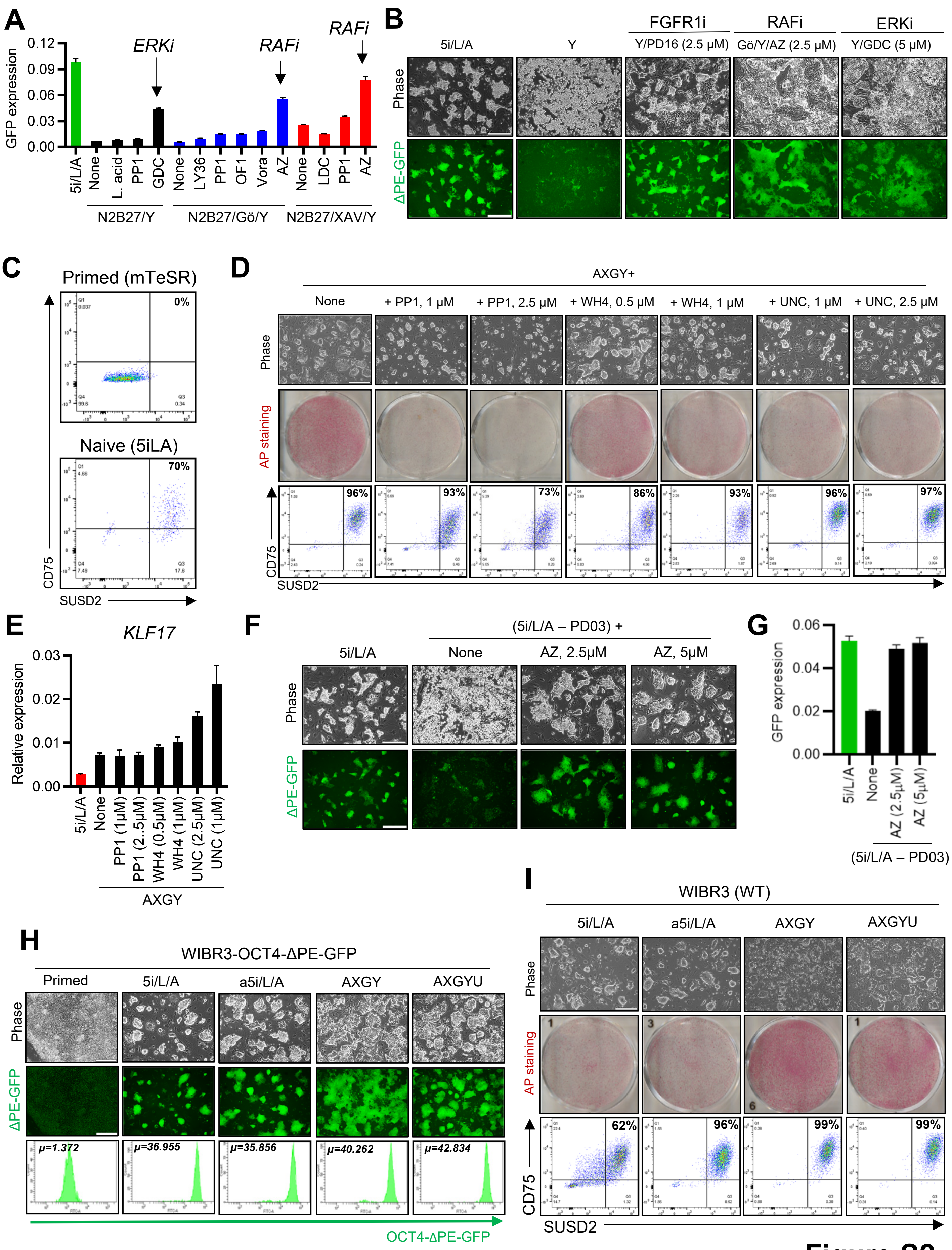

A
GFP expression
ERKi
RAFi
RAFi
5i/L/A
None
L. acid
PP1
GDC
None
LY36
PP1
OF1
Vora
AZ
None
LDC
PP1
AZ
N2B27/Y
N2B27/Gö/Y
N2B27/XAV/Y
B
5i/L/A
Y
FGFR1i
Y/PD16 (2.5 μM)
RAFi
Gö/Y/AZ (2.5 μM)
ERKi
Y/GDC (5 μM)
Phase
ΔPE-GFP
C
Primed (mTeSR)
0%
Naive (5iLA)
70%
CD75
SUSD2
D
AXGY+
None
+ PP1, 1 μM
+ PP1, 2.5 μM
+ WH4, 0.5 μM
+ WH4, 1 μM
+ UNC, 1 μM
+ UNC, 2.5 μM
Phase
AP staining
96%
93%
73%
86%
93%
96%
97%
CD75
SUSD2
E
KLF17
Relative expression
5i/L/A
None
PP1 (1μM)
PP1 (2..5μM)
WH4 (0.5μM)
WH4 (1μM)
UNC (2.5μM)
UNC (1μM)
AXGY
F
(5i/L/A – PD03) +
5i/L/A
None
AZ, 2.5μM
AZ, 5μM
Phase
ΔPE-GFP
G
GFP expression
5i/L/A
None
AZ (2.5μM)
AZ (5μM)
(5i/L/A – PD03)
H
WIBR3-OCT4-ΔPE-GFP
Primed
5i/L/A
a5i/L/A
AXGY
AXGYU
Phase
ΔPE-GFP
μ=1.372
μ=36.955
μ=35.856
μ=40.262
μ=42.834
OCT4-ΔPE-GFP
I
WIBR3 (WT)
5i/L/A
a5i/L/A
AXGY
AXGYU
Phase
AP staining
62%
96%
99%
99%
CD75
SUSD2

Figure S2

Figure S2. Efficacy of hit compounds during extended maintenance assays in naive hESCs [Related to Figure 2].

- **A.** Quantitative gene expression analysis for *GFP* in WIBR3 OCT4-ΔPE-GFP+ naive hESCs that were switched from 5i/L/A to the indicated conditions for two passages. In this experiment hit compounds were applied at 5 μM concentration. Error bars indicate mean ± SD of three technical replicates. Data are representative of two biological replicates.
- **B.** Phase (Top) and GFP fluorescence (Bottom) images of WIBR3 OCT4-ΔPE-GFP+ naive hESCs derived from primed hESCs using 5i/L/A and transferred to serum-free N2B27 medium supplemented only with ROCK inhibitor (N2B27/Y) or three alternative culture conditions that maintain GFP activity over two passages. Data are representative of two biological replicates. The scale bar depicts 250 μm.
- **C.** Flow cytometry analyses using antibodies against naive-specific cell surface markers CD75 and SUSD2 in H9 primed and naive hESCs cultured in mTeSR1 and 5i/L/A media, respectively. Data are representative of two biological replicates.
- **D.** Phase contrast (Top), Alkaline phosphatase staining (Middle), and flow cytometry analyses for naïve-specific cell surface markers CD75 and SUSD2 (Bottom) in H9 naive hESCs derived in 5i/L/A and switched to indicated conditions for two passages. The scale bar depicts 250 μm.
- **E.** Quantitative gene expression analysis for the naive-specific transcription factor *KLF17* in H9 naive hESCs for the samples shown in (D). Error bars indicate mean ± SD of three technical replicates.
- **F.** Phase (Top) and GFP fluorescence (Bottom) images of WIBR3 OCT4-ΔPE-GFP+ naive hESCs derived from primed hESCs using 5i/L/A and maintained for two passages in the absence of the MEK inhibitor PD0325901 (5i/L/A-PD03) or upon replacement of PD03 with the RAF inhibitor AZ628. Data are representative of two biological replicates. The scale bar depicts 250 μm.
- **G.** Quantitative gene expression analysis for *GFP* in the samples shown in (F). Error bars indicate mean ± SD of three technical replicates. Data are representative of two biological replicates.
- **H.** Phase images (Top), fluorescence images (Middle), and flow cytometry analysis (Bottom) in WIBR3 OCT4-ΔPE-GFP+ naive hESCs derived from primed hESCs using 5i/L/A and transferred to the indicated alternative naive maintenance conditions for two passages. The scale bar depicts 250 μm.
- **I.** Phase images (Top), Alkaline phosphatase (AP) staining (Middle), and flow cytometry analysis using naïve-specific CD75 and SUSD2 antibodies (Bottom) in wild-type WIBR3 naive hESCs that were derived from the primed state in 5i/L/A and maintained in the indicated naive maintenance conditions for two passages. The scale bar depicts 250 μm.

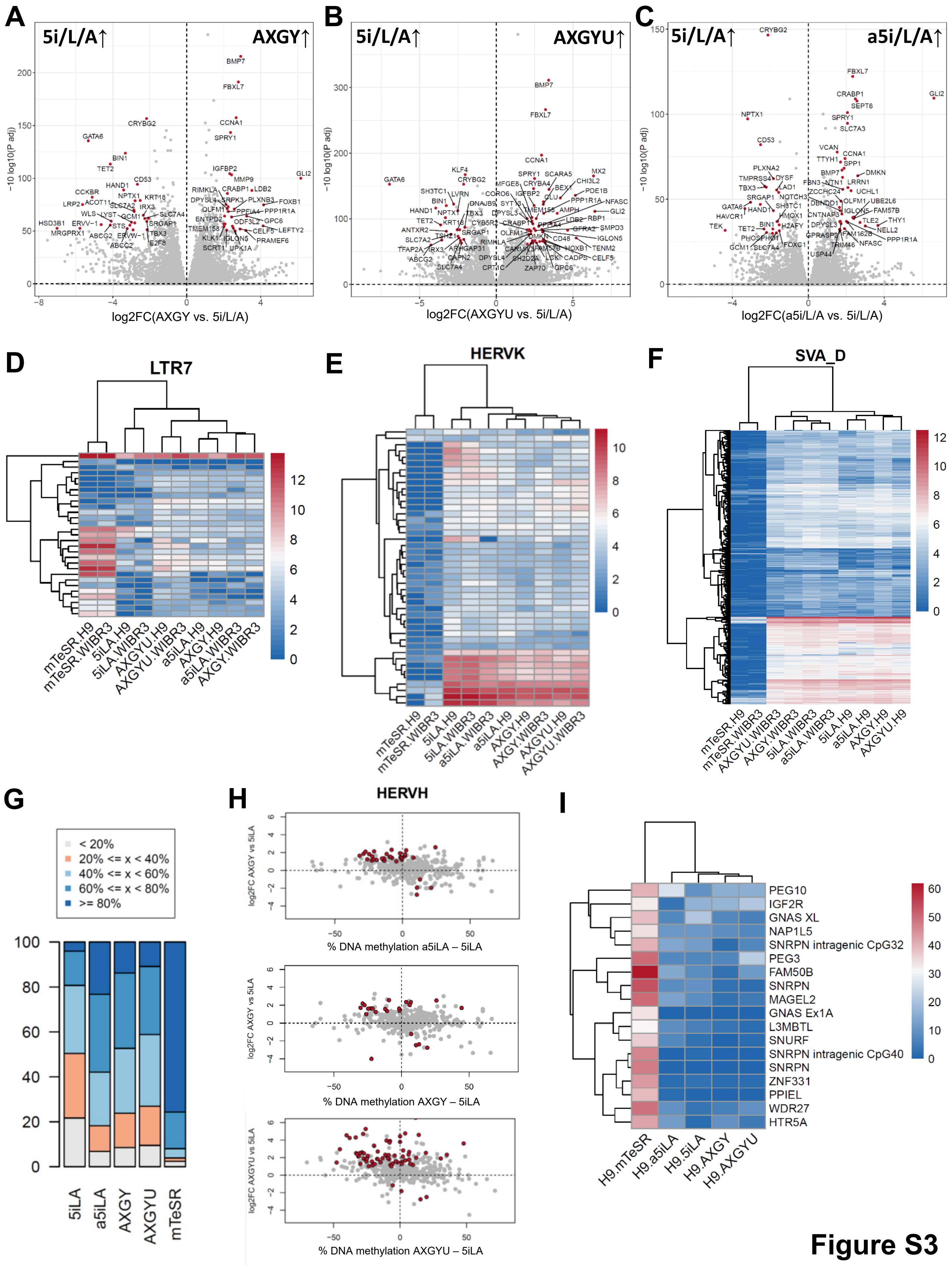
A
5i/L/A↑
AXGY↑
log2FC(AXGY vs. 5i/L/A)
−10 log10(P adj)
B
5i/L/A↑
AXGYU↑
log2FC(AXGYU vs. 5i/L/A)
C
5i/L/A↑
a5i/L/A↑
log2FC(a5i/L/A vs. 5i/L/A)
D
LTR7
E
HERVK
F
SVA_D
G
< 20%
20% <= x < 40%
40% <= x < 60%
60% <= x < 80%
>= 80%
5iLA
a5iLA
AXGY
AXGYU
mTeSR
H
HERVH
% DNA methylation a5iLA – 5iLA
% DNA methylation AXGY – 5iLA
% DNA methylation AXGYU – 5iLA
I
PEG10
IGF2R
GNAS XL
NAP1L5
SNRPN intragenic CpG32
PEG3
FAM50B
SNRPN
MAGEL2
GNAS Ex1A
L3MBTL
SNURF
SNRPN intragenic CpG40
SNRPN
ZNF331
PPIEL
WDR27
HTR5A
H9.mTeSR
H9.a5iLA
H9.5iLA
H9.AXGY
H9.AXGYU

Figure S3

Figure S3. Transcriptome and DNA methylome profiles of alternative naive hPSCs maintained without 2i [Related to Figure 3].

- **A.** Volcano plot showing differentially expressed genes (DEGs) in naive hESCs that were derived in 5i/L/A and subsequently maintained in AXGY vs. 5i/L/A. The x-axis plots log2 fold change and the y-axis plots significance as -log10(adj. p-value). Significant DEGs in both directions are labeled. Data represent two independent genetic backgrounds (H9 and WIBR3).
- **B.** Volcano plot showing differential expression in naive hESCs that were derived in 5i/L/A and subsequently maintained in AXGYU vs. 5i/L/A. The x-axis plots log2 fold change and the y-axis plots significance as -log10(adj. p-value). Significant DEGs in both directions are labeled. Data represent two independent genetic backgrounds (H9 and WIBR3).
- **C.** Volcano plot showing differential expression in naive hESCs that were derived in 5i/L/A and subsequently maintained in a5i/L/A vs. 5i/L/A. The x-axis plots log2 fold change and the y-axis plots significance as -log10(adj. p-value). Significant DEGs in both directions are labeled. Data represent two independent genetic backgrounds (H9 and WIBR3).
- **D.** Heatmap indicating expression of individual LTR7 integrants in H9 and WIBR3 primed hESCs and naive hESCs maintained in 5i/L/A or three alternative naive maintenance conditions.
- **E.** Heatmap indicating expression of individual HERVK integrants in H9 and WIBR3 primed hESCs and naive hESCs maintained in 5i/L/A or three alternative naive maintenance conditions.
- **F.** Heatmap indicating expression of individual SVA_D integrants in H9 and WIBR3 primed hESCs and naive hESCs maintained in 5i/L/A or three alternative naive maintenance conditions.
- **G.** Tile-based summary of genome-wide DNA methylation levels in H9 primed hESCs and naive hESCs maintained in 5i/L/A or three alternative naive maintenance conditions. This barplot shows the fraction of 1kB tiles with % methylation < 20%, 20 < x < 40 etc. for each condition.
- **H.** Correlation between expression of HERVH integrants (y-axis, log2FC in each alternative condition vs. 5i/L/A) and DNA methylation (x-axis, % DNA methylation in each alternative condition – 5i/L/A). Red dots indicate HERVH integrants that are significantly differentially expressed between the indicated conditions.
- **I.** DNA methylation levels at imprinted DMRs in H9 primed hESCs and naive hESCs that were derived in 5i/L/A and thereafter switched to three alternative naive maintenance conditions (a5i/L/A, AXGY or AXGYU). The coordinates of imprinted DMRs were defined in (Court et al., 2014; Theunissen et al., 2016).

A

Target	Compound name
ADRB2	ICI 118,551 hydrochloride
BMX	BMX-IN-1
CCR1	J113863
CHEK2	NSC 109555 ditosylate
EGFR	Erlotinib
ERBB	Sapitinib
GRM5	MPEP Hydrochloride
LXR	GSK2033
PTPN11	GS-493

B

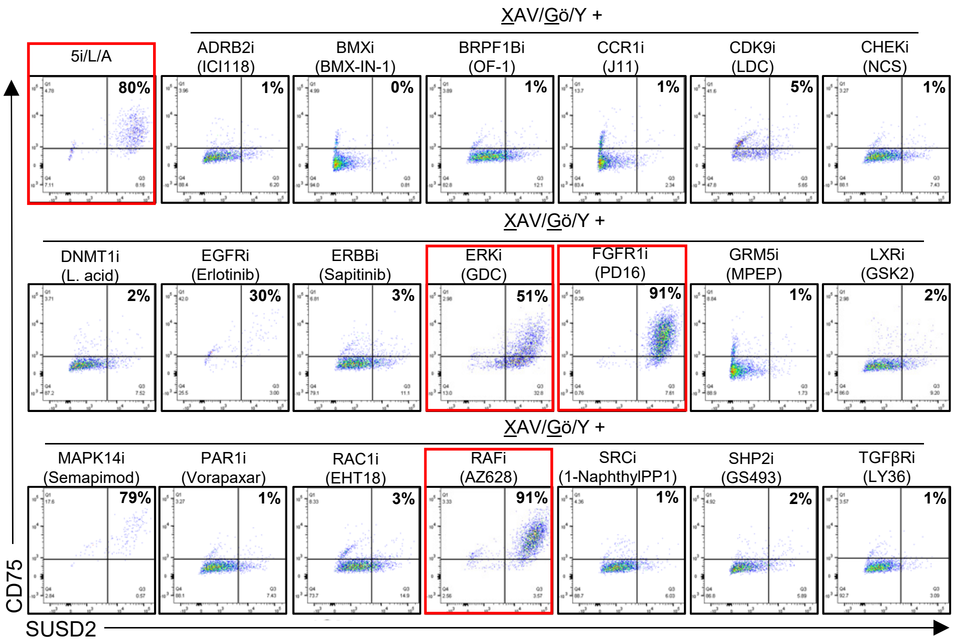

C

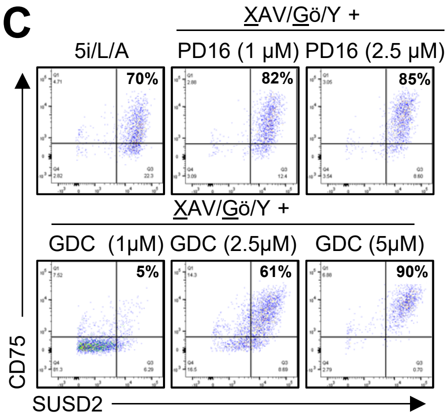

D

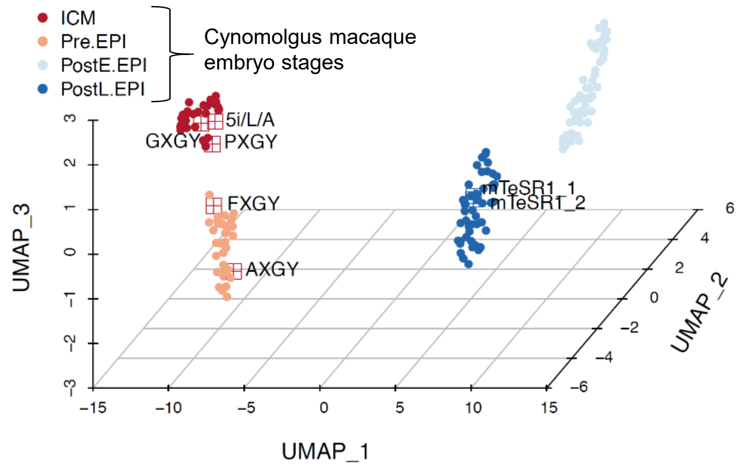

E

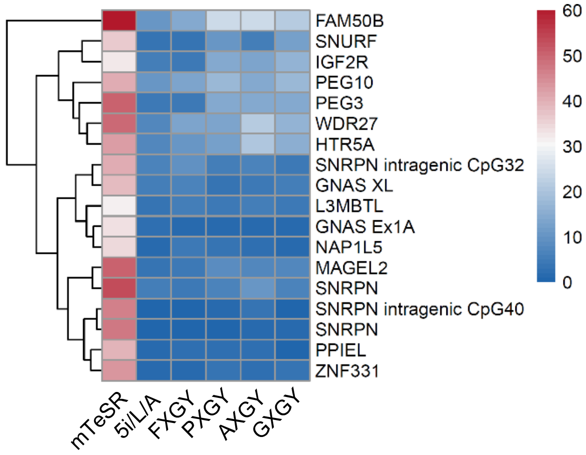

F

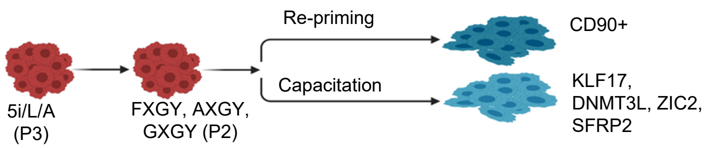

G

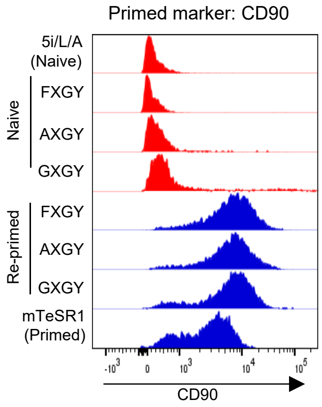

H

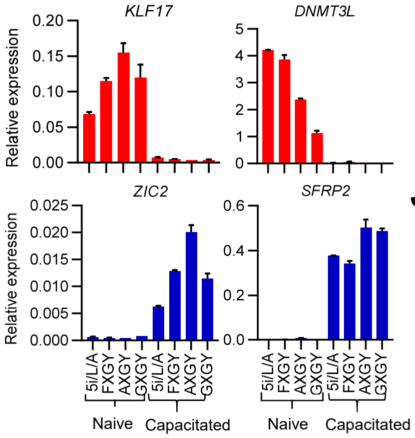

I

Media	Karyotype (Passage 10)
5i/L/A	5/20: Trisomy chr 8<br>7/20: Trisomy chr 3 and 8<br>3/20: Marker chr<br>5/20: Normal (20%)
FXGY	20/20: Normal (100%)
PXGY	3/20: Trisomy of chr 8<br>17/20: Normal (85%)
AXGY	20/20: Normal (100%)
GXGY	4/20: Trisomy of chr 8<br>16/20: Normal (80%)

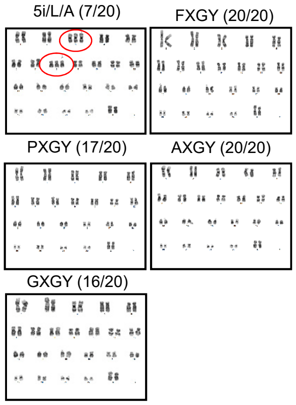

J

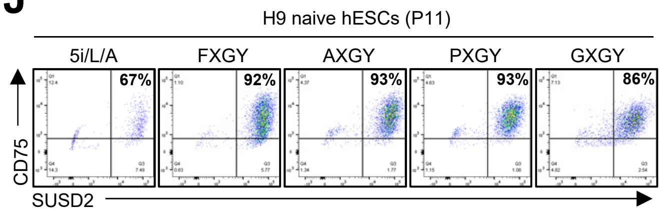

Figure S4

Figure S4. Validation screen with additional compounds and characterization of alternative naive maintenance conditions [Related to Figure 4]

A. List of alternative compounds for selected non-commercially available hit compounds shown in Table S2 and their corresponding targets.

B. Flow cytometry analysis with antibodies for naive-specific cell surface markers CD75 and SUSD2 to assess the ability of hit compounds to maintain naive hESCs in the presence of TNKS, PKC, and ROCK inhibitors (XAV/Gö/Y). 5i/L/A-derived H9 naive hESCs were switched to indicated conditions and maintained for two passages.

C. Titration experiment to determine the optimal concentrations of FGFRi (PD166866) and ERKi (GDC-0994) to maintain naive hESCs in XAV/Gö/Y. Flow cytometry analysis with antibodies for CD75 and SUSD2 was performed in 5i/L/A-derived H9 naive hESCs that were switched to the indicated conditions and maintained for two passages.

D. Uniform Manifold Approximation and Projection (UMAP) dimension reduction analysis of scRNA-seq data representing the inner cell mass (ICM), pre-implantation epiblast (Pre-EPI), early post-implantation epiblast (PostE.EPI), and late post-implantation epiblast (PostL-EPI) from Cynomolgus macaque embryo stages (Nakamura et al., 2017) compared to the alternative naive maintenance conditions described in Fig. 4C. Gene expression was compared to H9 mTeSR1 and 5i/L/A samples previously analyzed in Fig. 3 and an additional H9 mTeSR1 sample (mTeSR1_2).

E. DNA methylation levels at imprinted DMRs in H9 primed hESCs and naive hESCs that were derived in 5i/L/A and thereafter switched to four alternative naive maintenance conditions (FXGY, AXGY, PXGY, and GXGY). The coordinates of imprinted DMRs were defined in (Court et al., 2014; Theunissen et al., 2016). Data were compared to the H9 mTeSR1 sample previously analyzed in Fig. 3H.

F. Schematic representation of experiments used to assess the capacity of H9 naive hESCs that were maintained in the absence of MEK inhibition to undergo re-priming or capacitation into a formative pluripotent state using WNT inhibition (Rostovskaya et al., 2019).

G. Flow cytometry histogram indicating gain of the primed-specific cell surface marker CD90 at day 7 of re-priming of naive hESCs that were maintained in FXGY, AXGY, or GXGY.

H. Quantitative expression analysis for naive (*KLF17* and *DNMT3L*) and formative (*ZIC2* and *SFRP2*) markers following 10 days of capacitation. Error bars indicate mean ± SD of three technical replicates.

I. Summary of cytogenetic analyses of H9 naive hESCs maintained under various culture conditions (table) at P10 with representative images of predominant karyotypes for each culture condition. Chromosomal abnormalities in 5i/L/A are highlighted in red circles. For each sample 20 metaphase spreads were analyzed.

J. Flow cytometry analysis with antibodies for naive-specific cell surface markers CD75 and SUSD2 in H9 naive hESCs that were derived in 5i/L/A and switched to alternative maintenance conditions FXGY, AXGY, PXGY, and GXGY until P11.

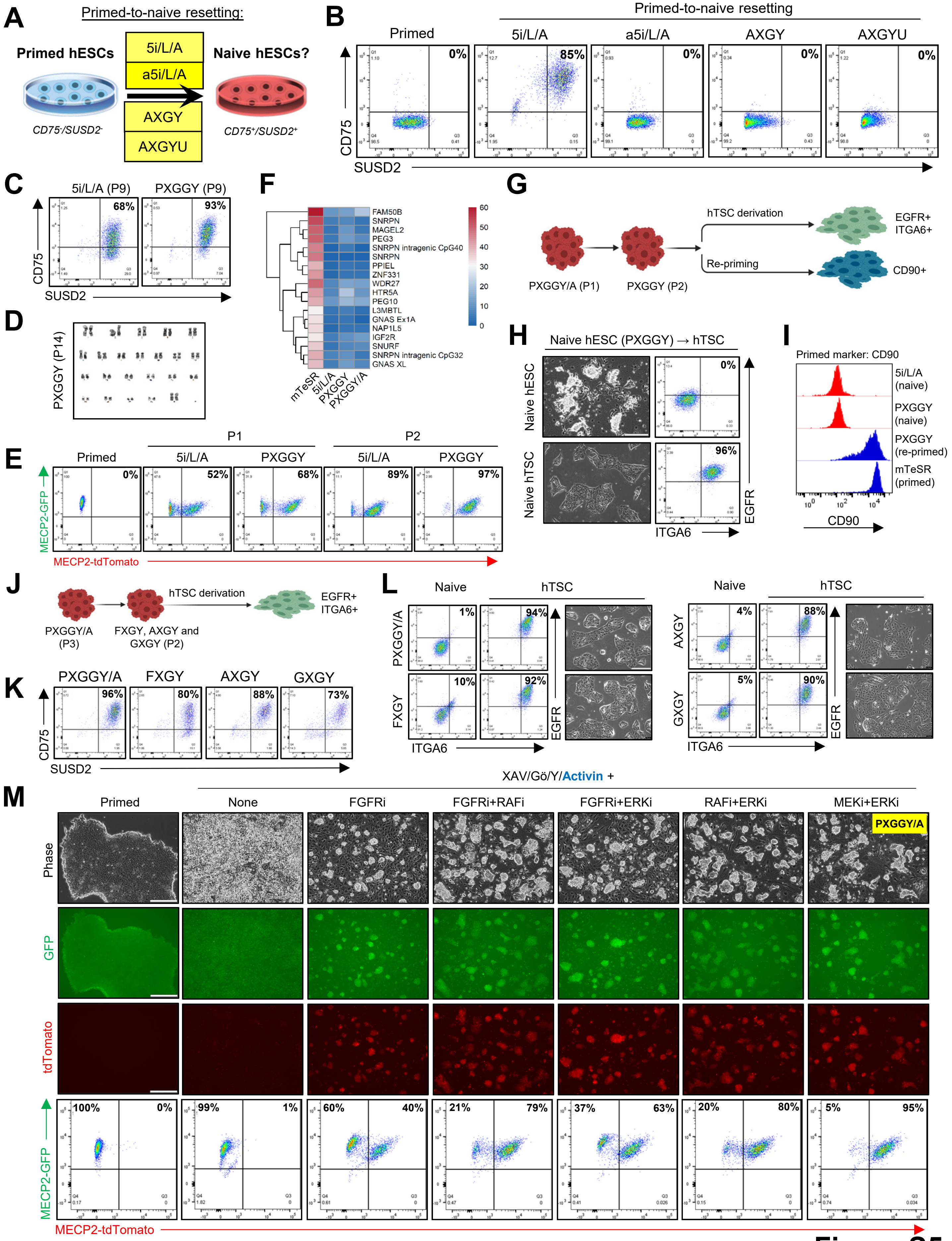
A
Primed-to-naive resetting:
Primed hESCs
5i/L/A
a5i/L/A
AXGY
AXGYU
Naive hESCs?
CD75-/SUSD2-
CD75+/SUSD2+
B
Primed-to-naive resetting
Primed
5i/L/A
a5i/L/A
AXGY
AXGYU
0%
85%
0%
0%
0%
CD75
SUSD2
C
5i/L/A (P9)
PXGGY (P9)
68%
93%
CD75
SUSD2
D
PXGGY (P14)
F
FAM50B
SNRPN
MAGEL2
PEG3
SNRPN intragenic CpG40
SNRPN
PPIEL
ZNF331
WDR27
HTR5A
PEG10
L3MBTL
GNAS Ex1A
NAP1L5
IGF2R
SNURF
SNRPN intragenic CpG32
GNAS XL
mTeSR
5i/L/A
PXGGY
PXGGY/A
G
hTSC derivation
Re-priming
EGFR+
ITGA6+
CD90+
PXGGY/A (P1)
PXGGY (P2)
H
Naive hESC (PXGGY) → hTSC
Naive hESC
Naive hTSC
0%
96%
EGFR
ITGA6
I
Primed marker: CD90
5i/L/A (naive)
PXGGY (naive)
PXGGY (re-primed)
mTeSR (primed)
CD90
E
P1
P2
Primed
5i/L/A
PXGGY
5i/L/A
PXGGY
0%
52%
68%
89%
97%
MECP2-GFP
MECP2-tdTomato
J
hTSC derivation
EGFR+
ITGA6+
PXGGY/A (P3)
FXGY, AXGY and GXGY (P2)
K
PXGGY/A
FXGY
AXGY
GXGY
96%
80%
88%
73%
CD75
SUSD2
L
Naive
hTSC
PXGGY/A
FXGY
1%
94%
10%
92%
EGFR
ITGA6
Naive
hTSC
AXGY
GXGY
4%
88%
5%
90%
EGFR
ITGA6
M
XAV/Gö/Y/Activin +
Primed
None
FGFRi
FGFRi+RAFi
FGFRi+ERKi
RAFi+ERKi
MEKi+ERKi
PXGGY/A
Phase
GFP
tdTomato
100%
0%
99%
1%
60%
40%
21%
79%
37%
63%
20%
80%
5%
95%
MECP2-GFP
MECP2-tdTomato

Figure S5

Figure S5. Examining the signaling requirements during primed-to-naive resetting [Related to Figure 5].

A. Experimental scheme to test the alternative naive maintenance conditions a5i/L/A, AXGY, and AXGYU during primed-to-naive conversion. Induction of naive pluripotency was assessed by flow cytometry using antibodies for the naive-specific cell surface markers CD75 and SUSD2 in H9 cells. Note that a5i/L/A consists of 5i/L/A media in which the MEK inhibitor PD03 is replaced with the RAF inhibitor AZ628.

B. Flow cytometry analyses using antibodies for the naive-specific cell surface markers CD75 and SUSD2 in H9 hESCs during primed-to-naive conversion in indicated conditions at the end of P1. Data are representative of two biological replicates.

C. Flow cytometry analysis using antibodies for the naive-specific cell surface markers CD75 and SUSD2 in H9 naive cells that were derived from the primed state in 5i/L/A or PXGGY at P9. Data are representative of two biological replicates.

D. Cytogenetic analysis at P14 for primed-to-naive conversion condition PXGGY showing a normal karyotype. 20 metaphase spreads were analyzed.

E. Flow cytometry analyses of naive WIBR3 *MECP2-GFP/tdTomato* reporter hESCs that were derived from the primed state in 5i/L/A or PXGGY at the end of P1 and P2.

F. DNA methylation levels at imprinted DMRs in naive hESCs that were converted in 5i/L/A or PXGGY/A at P5. We also examined naive hESCs that were derived in PXGGY/A and maintained for four additional passages in the absence of Activin. Data were compared to the H9 mTeSR1 and 5i/L/A samples previously analyzed in Fig. 4I. The coordinates of imprinted DMRs were defined previously (Court et al., 2014; Theunissen et al., 2016).

G. Schematic representation of experiments used to assess the capacity of H9 naive hESCs that were derived in PXGGY/A and maintained without Activin for two passages to undergo re-priming or differentiation into human trophoblast stem cells (hTSCs) (Dong et al., 2020).

H. Phase contrast images (Left) and flow cytometry analyses for hTSC-specific cell surface markers ITGA6 and EGFR (Right) of hTSCs derived from H9 naive hESCs that were isolated from the primed state in PXGGY/A and maintained without Activin for two passages. The scale bar depicts 250 µm.

I. Flow cytometry histogram indicating gain of the primed-specific cell surface marker CD90 during re-priming of naive hESCs that were derived from the primed state in PXGGY/A and maintained without Activin for two passages.

J. Schematic representation of experiments used to assess the capacity of H9 naive hESCs that were derived in PXGGY/A to undergo switching to alternative maintenance conditions and their differentiation into human trophoblast stem cells (hTSCs) (Dong et al., 2020).

K. Flow cytometry analysis using antibodies for the naive-specific cell surface markers CD75 and SUSD2 in H9 naive hESCs that were derived in PXGGY/A and switched to the alternative naive maintenance media (FXGY, AXGY, and GXGY) for two passages. These cells were subsequently used for hTSC differentiation.

L. Evaluation of the trophoblast potential of H9 naive hESCs that were derived from the primed state in PXGGY/A and subsequently maintained in alternative naive media without direct MEK inhibition. Flow cytometry analyses for hTSC-specific antibodies ITGA6 and EGFR (Left) and phase contrast images (Right) of hTSCs derived from indicated naive conditions. H9 cells shown in (K) were used for hTSC derivation. The scale bar depicts 250 µm.

M. Phase contrast images (Top), GFP and tdTomato fluorescence images (Middle), and flow cytometry analyses (Bottom) of WIBR3 *MECP2-GFP/tdTomato* reporter hESCs undergoing primed-to-naive conversion using various combinations of FGF pathway inhibitors in the presence of XAV/Gö/Y and Activin at the end of P1. The scale bar depicts 250 µm.
